# The Genetic Legacy of Multiple Beaver Reintroductions in Central Europe

**DOI:** 10.1371/journal.pone.0097619

**Published:** 2014-05-14

**Authors:** Christiane Frosch, Robert H. S. Kraus, Christof Angst, Rainer Allgöwer, Johan Michaux, Jana Teubner, Carsten Nowak

**Affiliations:** 1 Conservation Genetics Group, Senckenberg Research Institute and Natural History Museum, Gelnhausen, Germany; 2 Centre Suisse de Cartographie de la Faune (CSCF), Neuchâtel, Switzerland; 3 Büro für Ökosystemforschung, Mühlacker, Germany; 4 Unité de Recherches Zoogéographiques, Institut de Zoologie, Liège, Belgium; 5 Naturschutzstation Zippelsförde, Zippelsförde, Germany; 6 Biodiversity and Climate Research Centre (BiK-F), Frankfurt am Main, Germany; Tuscia University, Italy

## Abstract

The comeback of the Eurasian beaver (*Castor fiber*) throughout western and central Europe is considered a major conservation success. Traditionally, several subspecies are recognised by morphology and mitochondrial haplotype, each linked to a relict population. During various reintroduction programs in the 20th century, beavers from multiple source localities were released and now form viable populations. These programs differed in their reintroduction strategies, i.e., using pure subspecies *vs.* mixed source populations. This inhomogeneity in management actions generated ongoing debates regarding the origin of present beaver populations and appropriate management plans for the future. By sequencing of the mitochondrial control region and microsatellite genotyping of 235 beaver individuals from five selected regions in Germany, Switzerland, Luxembourg, and Belgium we show that beavers from at least four source origins currently form admixed, genetically diverse populations that spread across the study region. While regional occurrences of invasive North American beavers (n = 20) were found, all but one *C. fiber* bore the mitochondrial haplotype of the autochthonous western Evolutionary Significant Unit (ESU). Considering this, as well as the viability of admixed populations and the fact that the fusion of different lineages is already progressing in all studied regions, we argue that admixture between different beaver source populations should be generally accepted.

## Introduction

After massive population bottlenecks and regional extinctions through active human persecution until the early 20^th^ century several large and medium-sized mammals such as brown bear, lynx, wolf, wisent, or beaver currently show a stunning comeback throughout Western and Central Europe [1], [2]. In some cases reestablishment of these species resulted from natural long-distance dispersal. In others however, human-assisted reintroduction projects have been undertaken and have proven successful in restoring species to areas where they had become regionally extinct e.g. [3]–[6]. In Central Europe, for instance, reintroduction projects were initiated during the 20^th^ century for numerous large mammal species [3]–[6]. Several of these projects proved successful and restocked formerly unoccupied areas. For some species such as Eurasian beavers (*Castor fiber* Linneaus 1758), the high success rates of reintroductions, even with low founder numbers, led to a “reintroduction boom”, with projects occurring across Germany as well as neighbouring regions.

**Figure 1:**
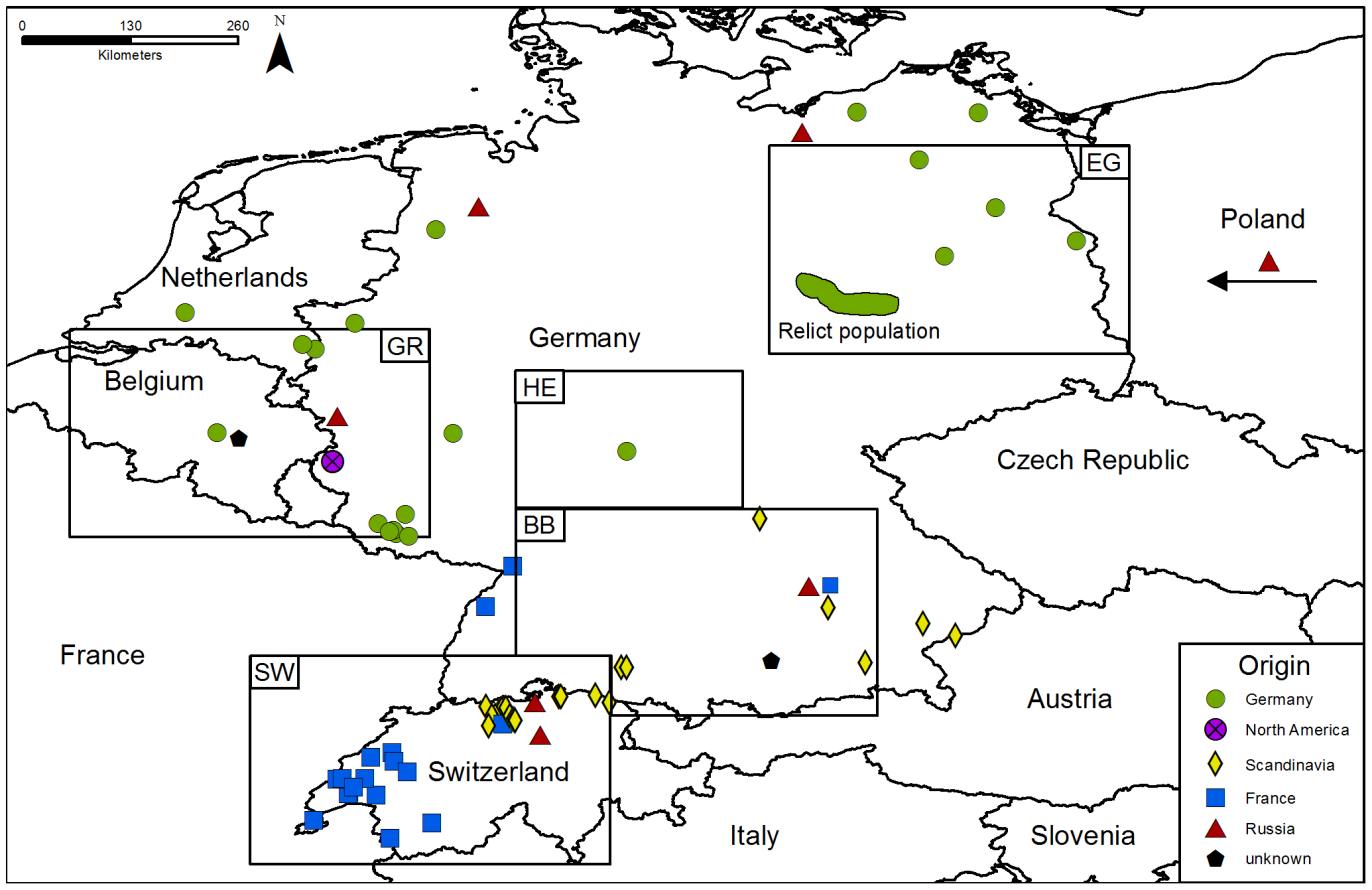
Reintroduction map of beavers in Germany, Belgium, the Netherlands and Switzerland. Boxes represent five different investigated areas. Symbols represent reintroduction locations and show from which population beavers were relocated. Detailed information of the reintroduction history in the five regions is provided in the Text S1.

Beavers were anthropogenically reduced to only few scattered relict populations in Eurasia [7] by the beginning of the 20^th^ century. Various subspecies were initially defined based on the geographic location of their refugia and subtle morphological differences [8]. Later it was found that this massive bottleneck reduced genetic diversity in the relict populations severely [9]. Therefore, only a single or few mitochondrial control region (CR) haplotypes, which were all specific for each relict population, were preserved [10], [11]. These matched designated subspecies and could be assigned to two major mtDNA clades [10], which were consequently proposed to form an eastern and a western Evolutionary Significant Unit (ESU) *sensu* Moritz *et al*. [12] (note, however, that ESU delineation may have been flawed in Durka *et al*. [10] because no nuclear genetic information was presented). In our study region, including Germany, Switzerland and Belgium, >50 reintroductions released an unknown number of beavers of various origins, including relict populations of the presumed western ESU [10]: *C. f. albicus* Matschie 1907 (relict population in Germany), *C. f. galliae* Geoffroy 1803 (relict population in France), *C. f. fiber* L. 1758 (relict population in Norway); as well as beavers from the Voronezh breeding station in Russia (*C. f. orientoeuropaeus* Lavrov 1981), so far presumed to belong to the eastern ESU [13] (Fig. 1). Four additional subspecies of the eastern ESU *C. f. birulai* Serebrennikov 1929 (relict population in China and Mongolia), *C. f. tuvinicus* Lavrov 1969 (relict population in West-Siberia), *C. f. pohlei* Serebrennikov 1929 (relict population in Middle Siberia), and *C. f. belorussicus* Lavrov 1981 (relict population in Belarus) are described [7], [10], but are not recorded to be reintroduced within our study area.

While several reviews have attempted to reconstruct the complex reintroduction history of beavers in Eurasia e.g. [7], [14]–[16], no subsequent approach or comprehensive super-regional plan has been implemented for beaver reintroductions in Europe [13]. Halley [13] identified three basic strategies concerning the choice of source populations: (i) use of the geographically closest beaver lineage ( = relict population), (ii) mixture of animals from two or three western lineages; and (iii) release of *C. fiber* individuals of multiple origins, regardless of ESU assignment. In Western Europe, not only were all three schemes applied, but the underlying philosophies of each beaver reintroduction project also differed fundamentally. Several reintroductions were not monitored and the literature is scattered or inconsistent. Additionally, North American beavers (*C. canadensis* Kuhl 1820) that potentially escaped from captivity may have contributed to recolonisation [16]. This release of species and presumed subspecies of beavers from various Holarctic origins, although questionable in regard to IUCN guidelines, formed a natural experiment regarding the grades of admixture between lineages and the potential effects of local adaptation, inbreeding (presumably dominant in non-admixed relict populations and their descendants) and outbreeding depression (potentially occurring in admixed populations originating from different lineages within or across ESUs).

In order to unravel the reintroduction and dispersal history of beavers in Germany and adjacent regions, we sampled tissue from animals found dead in the wild as well as noninvasively collected hair samples. Special emphasis was placed on known regions where beavers originate from different source populations. Mitochondrial CR haplotype analysis and nuclear microsatellite markers were applied in five study regions to reveal the degree of potential admixture between beavers from different origins. Beaver populations in these regions differ by origin of source animals as well as time since reintroduction, thus providing snapshots into different time windows of potential admixture within contact zones. Specifically, we addressed the following four questions.

- Which species (native *C. fiber*, invasive *C. canadensis*) and relict populations (“subspecies”) contribute to the present distribution of beavers in the study region?
- Have the different beaver source populations recently fused and formed admixed populations with elevated levels of genetic diversity?
- Is admixture ongoing and will it potentially lead to the disappearance of the classically recognised “subspecies” in the study region, including the relict population of Elbe beaver (*C. f. albicus*) in the study region?

We interpret our data with regard to potential effects of inbreeding and outbreeding depression as well as the delineation of ESUs and the categorisation of presumed subspecies. We place specific emphasis on the potential consequences of population admixture for species conservation and population management.

## Methods

### Study Regions

We investigated five zones of secondary contact between different reintroduced populations in Germany, Luxembourg, Belgium, and Switzerland (Fig. 1). In these regions different source populations co-occur in spatial vicinity, forming potential intraspecific hybrid zones.

In the German federal state Hesse (Region I; HE) we investigated the potential hybrid zone between individuals from the Spessart Mountains and the population in southern Hesse and northern Bavaria (n = 42). Eighteen individuals from the German relict population (*C. f. albicus*) were reintroduced to the Hessian Spessart Mountains in 1987–1988, while the southern part of this region is naturally colonised. Furthermore, we investigated a region in Eastern Germany (federal state Brandenburg and the border area between Saxony-Anhalt and Saxony; Region II; EG) including the border to Poland (n = 53). Few individuals of the German relict beaver population *C. f. albicus* survived in region EG and the population was supported by several successful reintroductions of individuals from the Elbe river system since 1935. Since 1974 beavers from the Polish beaver farm in Popielno (founded with beavers originating from the beaver farm in Voronezh, Russia) were reintroduced to Poland and currently disperse to Germany where we investigated the admixture between the German and the Polish population. In the German federal states Bavaria and Baden-Württemberg (Region III; BB) beavers of different origins (Scandinavia, Russia, France) were reintroduced since 1966 (n = 64). Region IV (SW) comprises Switzerland including samples from the border to France and from Baden-Württemberg. Here, we analysed 32 samples from the contact zone where 141 individuals from the French relict population (*C. f. galliae*), from Norway (*C. f. fiber*) and from Voronezh, Russia, were reintroduced between 1956–1977. Additionally, we analysed 44 samples from Belgium, Luxembourg and the German federal states Rhineland-Palatinate and Northrhine-Westphalia, also known as *The Greater Region* (GR; Region V), where beavers from the Elbe relict population, from Poland (wild catches and farmed beavers from Popielno) and Bavaria were reintroduced since 1981. Since 2006 the occurrence of *C. canadensis* is recorded for Luxembourg, Rhineland-Palatinate and Belgium. Detailed information for every region is provided in the Text S1.

### Sampling and DNA Extraction

No beavers were trapped or killed for this study. Noninvasive hair samples were collected using barbed wire traps without animal handling (Region HE, GR). Normally, one tuft of hair containing 20–30 wool hairs and 2–5 guard hairs was found at the traps. Beaver hair traps were set up in a height such that only adult beavers were sampled and barbed wire was sufficiently flexible as to avoid scratching animal skin (no signs of blood were ever observed during our hair trapping campaigns), and the wooden sticks to which the wire was attached was sufficiently loosely anchored to the ground that potential entanglement or suffocation of beavers (or other by-passing animals) was impossible (we never found injured or dead animals at the hair traps). Thus, no beavers or other wildlife were harmed following the placement of the hair traps and in the process of collecting the hair samples. Tissue samples of *C. fiber* originated from animals found dead, due to traffic mortality, illness; or they died of old age (Region HE, EG, BB, SW, GR and Russian reference samples). Culling was carried out by regional authorities during coordinated beaver management (13 individuals in region BB). Tissue samples of the invasive *C. canadensis* were obtained during a sterilisation programme by the federal state Rhineland-Palatinate with the aim to prevent the spreading of the North American beaver (Region GR). After sterilisation *C. canadensis* individuals were transferred to their original territory or zoos. Detailed information including sample location, sample material, and name of the collector providing the sample, as well as information about the means of sample acquisition for every sample and the approving authorities can be checked in Table S1. In total, 178 tissue samples and 57 collected hair samples were used in this study. Of these, 80% were collected between 2008 and 2012, 17% are up to 10 years old, and 3% are older than 10 years (see Tables S1 and S2). For comparison we had access to three samples from Kirov, Russia, as numerous beavers from Russia were reintroduced in Central Europe.

For DNA extraction from tissue we used the Qiagen Blood and Tissue Kit following the manufacturer’s instructions and diluted DNA to 6.5 ng/µl for further analyses. From areas where insufficient numbers of tissue samples were available we collected hair samples with barbed wire traps [17]. In the years 2011/2012 we placed 61 barbed wire traps in HE, which were inspected 412 times in total. Hair samples were stored in filter paper envelopes with silica gel packs at room temperature. In 34.7% of the inspections of the barbed wire traps we found an adequate number of hairs for genetic analysis. We exclusively used hair samples from barbed wire traps set up in different beaver territories to minimise the risk of sampling individuals twice. Another 35 hair samples were plucked from caught animals. We analysed 57 hair samples for this study using 5–10 hairs and processed these with the Qiagen Investigator Kit as per manufacturer instructions. Hair samples were prepared in a separate laboratory room dedicated to handling low amounts of DNA and considering the standard routines for non-invasive sample treatment to avoid contamination [18]. The consensus genotype was constructed by hand based on three independent PCR replicates. Allelic dropout occurred at 3% (range 0–12%) and false alleles at 1% (range 0–6%; Table S3).

### Mitochondrial DNA Amplification and Analysis

We sequenced a part of the hyper-variable domain of the control region of mitochondrial DNA using the oligonucleotides 1F (5′-AATTACTTTGGTCTTGGTAAACC-3′) and 6R (5′-GCCCTGAAGTAAGAACCAGATG-3′) Horn [19]. Polymerase chain reactions (PCR) took place in a final volume of 15 µl and contained 0.1 U of *Taq* polymerase (New England Biolabs), 1.5 µL of 10× *Taq* polymerase buffer, 1.8 mM MgCl_2_, 0.2 mM each dNTP, 0.25 mg/ml BSA, and 0.3 µM each primer. We used the following thermal cycling parameters: 5 min at 94°C, 40 PCR cycles (55 s at 94°C, 45 s at 54°C, 45 s at 72°C) plus 10 min at 72°C. PCR products were purified using Exo-Sap-it (Affymetrix). We sequenced the amplicons on a 3730 DNA Analyzer (Applied Biosystems) in both directions with Big-Dye Terminator v3.1 chemistry (ABI), aligned the sequences with ClustalW 1.83 [20] and calculated basic sequence analyses and pairwise genetic distances in MEGA 5.10 [21].

A phylogenetic network was constructed in TCS 1.21 [22] by statistical parsimony, treating alignment gaps as fifth state. We combined the beaver haplotypes obtained in this study with 12 additional haplotypes downloaded from GenBank (NCBI). New mtDNA sequences were deposited in GenBank (r_2_ = KF731635; r_3_ = KF731636; e = KF731637; c = KF731638).

### Microsatellite Analysis

We initially analysed 25 microsatellites (see Table S4) originally identified in Castor. The five markers from Pelz-Serrano *et al.* [23] as well as Cca13 and CF48 [24], [25] were excluded from the analyses due to suboptimal amplification. The remaining 19 markers were grouped in four multiplex PCR reactions. PCR was performed using Qiagen master mix in 10 µl reactions including 3.6 µl DNA extract, 1.5 mM MgCl_2_, 0.2 mM each dNTP, 0.2 µM each primer, 0.25mg/ml BSA, and 0.5 U/µl HotStar *Taq*-Polymerase (Qiagen). Fragments were amplified under the following cycle conditions: 15 min at 95°C, 45 cycles of 30 s at 94°C, 90 s annealing at 50°C, 60 s at 72°C, and final elongation of 30 min at 72°C. For all PCR reactions positive and negative controls were included. Hair samples were analysed in three replicates. We measured amplicon fragment length on an ABI 3730 DNA Analyzer (Applied Biosystems), using deionized formamide and Genescan size standard LIZ500 (Applied Biosystems), and analysed the raw data with Genemarker 1.6 (SoftGenetics). Six Loci showed inconsistent results with strong deviations from Hardy-Weinberg equilibrium (HWE) and were thus excluded from all further analyses. All following analyses are based on 13 microsatellites: Cca4, Cca8, Cca13, Cca18, CF05, CF06, CF07, CF19, CF31, CF32, CF33, CF41, CF44.

### Genetic Diversity

Genotyping errors (allelic dropout, false alleles) of hair samples were calculated using Gimlet 1.3.3 [26]. We calculated observed and expected heterozygosity at each locus and tested for deviations from HWE for each of the five regions and their sub-populations with Arlequin 3.5 [27] using the analog to Fisher’s exact test for arbitrary table size [28] (1,000,000 Markov chain steps, 100,000 dememorisation steps).

We investigated two measures for allelic richness: the observed, uncorrected allelic richness (plain counts of alleles per locus and per study group) and corrected allelic richness (to prevent sample size bias) as determined by rarefaction (HP-Rare 1.1) [29]. We compared allelic richness among regions by correcting to a sample size of 27 (the smallest sample size among regions and loci). Within regions HE, EG, BB and SW individuals were sorted into two sub-populations according to Structure with *K* = 2 and admixed individuals (q<0.8) were excluded. Here, rarefaction was applied according to the minimum sample size per sub-population within each region (HE = 12; EG = 15; BB = 12; SW = 10). Allelic richness for *C. canadensis* was not corrected for sample size because all individuals were sampled within the same region; no comparison to other regions is needed. For more details and calculations per marker see Table S5. Means of corrected allelic richness of both sub-populations for each region were compared by a Welch t-test, function *t.test()*, after we tested the data for normality using the Shapiro-Wilk Test, function *shapiro.test()*, in R (R Development Core Team 2009). None of the data sets significantly deviated from normality when applying sequential Bonferroni correction (12 tests) [30]. Region GR represents a special case as this population consists of a complex species/subspecies structure and cannot simply be sorted in two groups so that only the overall data is shown.

### Genetic Admixture Analysis

#### Structure

Structure v2.3.3 [31] was used to infer population structure and to assign individuals to *K* populations. We used the admixture model with correlated allele frequencies and ran the software for 1,000,000 steps (including 300,000 steps burn-in). We tested a range of *K* from 1 to 20, with 10 replicates for each *K* for the complete data set as well as for the data set without *C. canadensis*. For analyses within the five regions we tested a range of *K* from 1–10 (10 replicates per *K*). The most likely *K* was inferred using Evanno *et al.*’s [32] method in Structure Harvester [33].

#### DAPC

In addition to Structure we used Discriminant Analysis of Principal Components (DAPC) [34] from adegenet [35] version 1.3–8 in R to identify and describe clusters of genetically similar individuals. Using the function *find.clusters*, we determined the most likely number of genetic clusters in each study group, using all principal components (PCs). To calculate the assignment probability of a beaver to each of these clusters we determined the optimal number of principal components (PCs) as advised in the manual. To avoid unstable assignments of individuals to clusters, we retained for every analysis a number of PCs equaling the respective sample size of the group to analyse divided by three, but used all discriminant functions in a preliminary DAPC run. We then used the *optim.a.score* function with 20 simulations to determine the optimal number of PCs, and a final DAPC was subsequently carried out with this optimal number of PCs.

#### NewHybrids

NewHybrids [36] estimates the probability of assignment of each individual to a particular hybrid generation or category (i.e., parental groups, F_1_ hybrids, F_2_ hybrids, backcrosses). We tested the four regions HE, EG, BB, and SW individually with a burn-in period of at least 200,000 repetitions followed by a run with minimally 800,000 steps for genetic admixture and excluded region GR because the two occurring species in this area, *C. canadensis* and *C. fiber*, do not hybridise [37].

## Results

### Mitochondrial Haplotype Analysis

**Figure 2:**
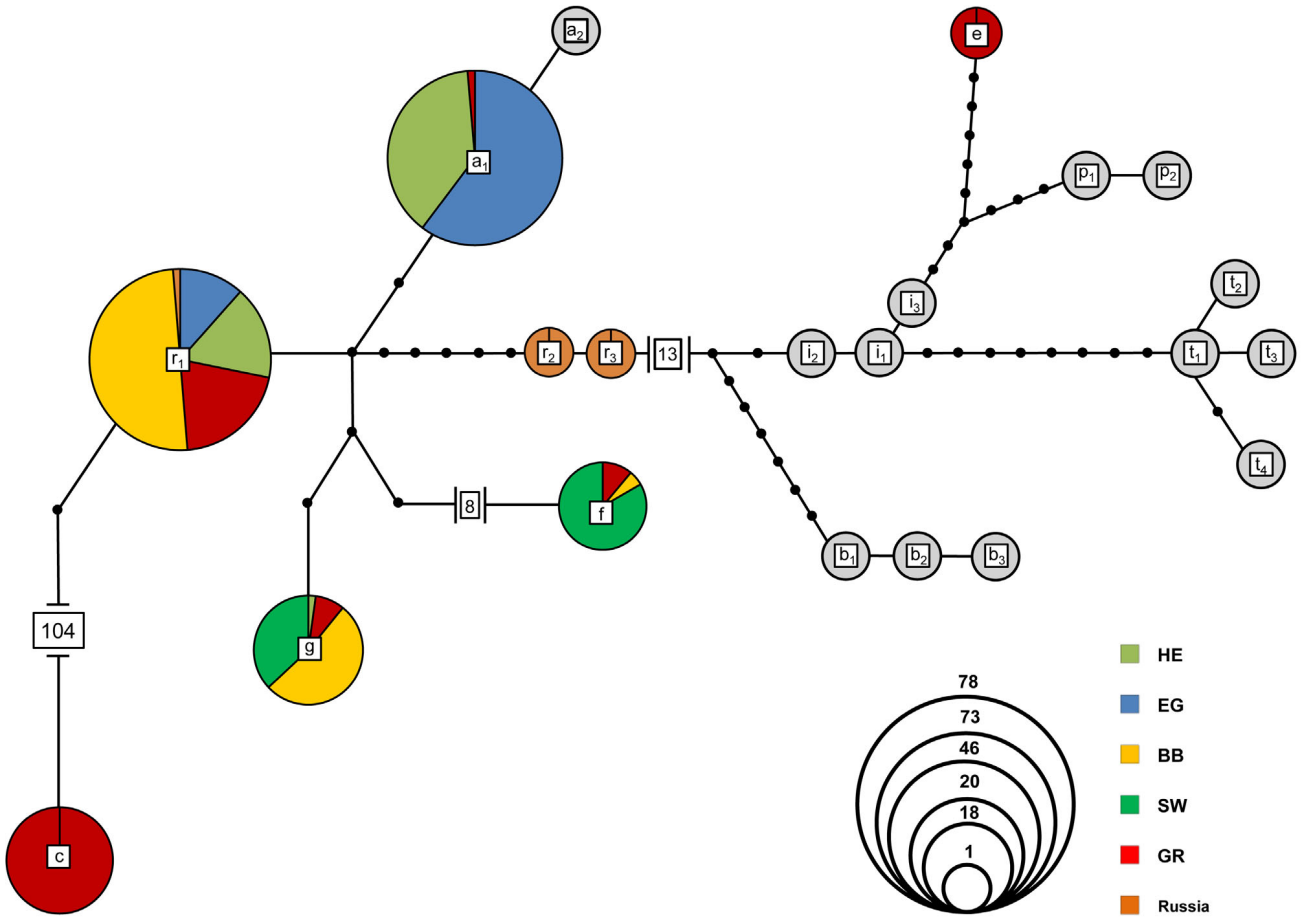
TCS network of 235 analysed beavers using a 487–489 bp fragment of the mitochondrial CR. Eight haplotypes were detected in this study (coloured) and 13 additional haplotypes originate from GenBank (grey): DQ088701 (a_2_), AY623634 (b_1_), AY623633 (b_2_), AY623632 (b_3_), AY623642 (i_1_), AY623641 (i_2_), AY623643 (i_3_), AY623635 (p_1_), AY623636 (p_2_), AY623637 (t_1_), AY623638 (t_2_), AY623639 (t_3_), AY623640 (t_4_). Sizes of circles are proportional to the number of samples bearing the haplotype. Colours of circles indicate the origin of the samples.

Sequencing of 57 hair samples and 178 tissue samples (plus three reference samples from Kirov, Russia) yielded eight CR haplotypes. Four of those were previously described: *C. f. galliae* DQ088703 (g), *C. f. fiber* DQ088702 (f) and *C. f. albicus* DQ088700 (a_1_) [10] plus haplotype r_1_ JF264887 [19]. Two of our haplotypes from Russia (r_2_ and r_3_), the only haplotype of the eastern lineage (e) and the haplotype of *C. canadensis* were not described so far (Table S6). Genetic pairwise distances between *C. canadensis* and *C. fiber* were on average 25%. Within the 487–489 bp fragment 44 variable sites were present among all *C. fiber* samples. Haplotypes group into two divergent beaver lineages (Fig. 2), namely the western and eastern lineage as already observed in [10]. Interestingly, all but one *C. fiber* samples, including the three samples from the Russian Voronezh region, clustered within the western ESU.

### Nuclear Genetic Analysis

#### Complete data set

All 13 microsatellites of the final marker set were polymorphic for *C. fiber* (3–7 alleles per locus). The nine microsatellites (CF32, Cca18, Cca13, CF33, Cca4, Cca8, CF6, CF31, CF19) were also polymorphic for *C. canadensis* (2–4 alleles). Together, in both species we found 75 alleles of which 12 were exclusively found in *C. canadensis*. Microsatellite genotype data is provided in an additional file (Table S8).

**Figure 3:**
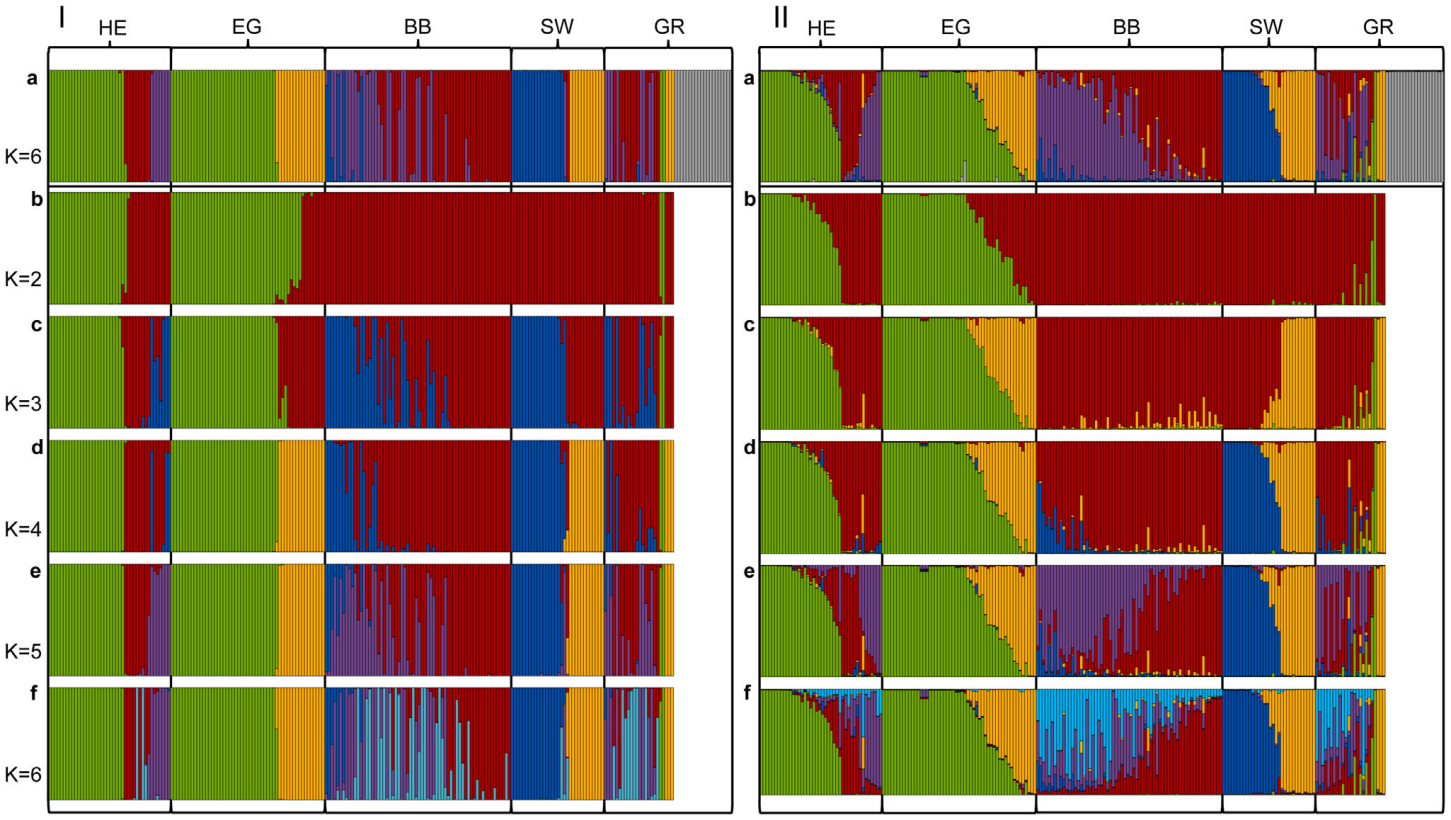
Results of DAPC (I) and Structure (II) analyses. Panel a represents the results with *K* = 6 for *C. fiber* and *C. canadensis* samples (grey), b-f show results of the *C. fiber* samples for *K* = 2–6. Samples are sorted according to the five investigated regions.

**Figure 4:**
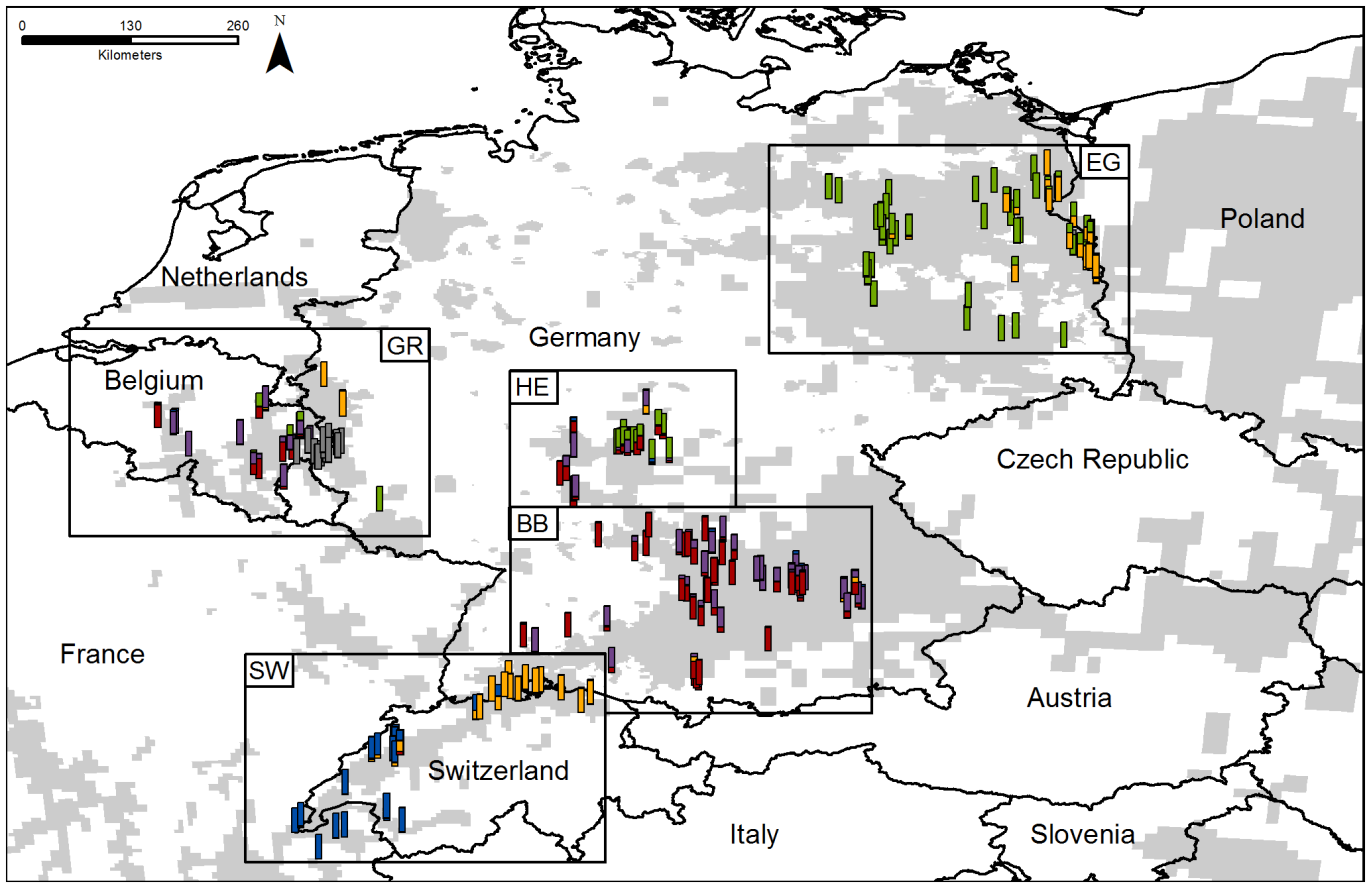
Map of Germany and adjacent countries. Shaded background displays the current distribution range of beavers in the study area (based on [6], [16], [19], [52]). Every bar shows the origin of a sample. Colours of bars are according to Structure assignments (*K* = 6) and Fig. 3IIa.

The most likely number of genetic clusters for the whole data set was *K* = 6 (Figs. 3 and 4). This result was stable for DAPC and Structure analyses and *C. canadensis* formed a clearly separated cluster (grey, Fig. 3a). Within regions, the proportion of individual genetic admixture was generally higher when genotypes were analysed with Structure. DAPC was more decisive with respect to membership coefficients of individuals to specific genetic clusters, such that more beavers were assigned with high posterior probability (>0.8) to a genetic cluster in DAPC than in Structure. For *K* = 6 (including all analysed 235 beavers) DAPC assigned 228 individuals (97.02%) clearly to a group whereas Structure assigned 169 individuals to one of the populations (71.91%).

To display all sub-structuring in the data set of *C. fiber* we compared the runs *K* = 2–6 for DAPC (Fig. 3 Ib–f) and Structure (Fig. 3 IIb–f). An analysis with *K* = 2 differentiates between the German relict beaver lineage *C. f. albicus* (green; Fig. 3b) in Region HE, Region EG and Region GR *vs.* all other specimens (red). When analysing the data with *K* = 3 the third group represents individuals with a French ancestry of *C. f. galliae* (blue; Fig. 3c) in DAPC whereas Structure splits off a third group including samples from the Rhine watershed in Region SW but also from individuals of Region EG which likely dispersed from Poland to north-eastern Germany (yellow; Fig. 3d). For *K* = 4 the overall picture is consistent between DAPC and Structure. The value of *K* with the highest likelihood and consistency between runs for the data set of *C. fiber* (excluding *C. canadensis*) was *K* = 5 (Fig. 3e). Here, the red group is subdivided in two (red and purple; having two private alleles). *C. f. albicus* from HE and BB also form a cluster (green; one private allele). The blue group is of French origin (two private alleles). The yellow cluster represents Swiss and EG beavers; in this eastern group we found an additional private allele. The higher *K* models did not reveal deeper, biologically meaningful structure and also no split between the geographically widely separated Rhine watershed in Switzerland and eastern Brandenburg in EG.

### Regional Admixture Zones

#### Region HE (Fig. 5)

We found two main CR haplotypes: a_1_, representing the reintroduced *C. f. albicus* population and r_1_, likely stemming from reintroduced Russian individuals, as well as the individual HE29 carrying the French haplotype g. DAPC clearly separated individuals with haplotype a_1_ (except HE28) and a group with r_1_/g but Structure and NewHybrids identified a number of genetically admixed individuals (HE20–28). The individuals translocated to Hesse in 1987–1988 still reside in the region of original location in the Hessian Spessart Mountains. However, the population of southern Hesse spreads northwards to central and eastern Hesse (HE36), with a genetic impact already detectable in the core zone of the reintroduction area (see, e.g., HE23, HE24, HE26 and HE28).

#### Region EG (Fig. 6)

In north-eastern Germany we found two haplotypes: a_1_, the indigenous German haplotype but also r_1_ (Russian haplotype). DAPC inferred two clusters roughly according to geography, namely a western (green) and an eastern part (yellow). Individuals EG37 and EG38 were not clearly assigned to either population. In Structure slightly more individuals had an intermediate genotype. NewHybrids, in contrast, suggested substantial admixture between Region EG and the immigrating Polish individuals.

#### Region BB (Fig. 7)

In Bavaria individuals from multiple origins were reintroduced and also dispersed far into Baden-Württemberg. We detected individuals with haplotypes of three origins: Scandinavia (*C. f. fiber*; n = 1, f), France (*C. f. galliae*; n = 24, g) and Russia (n = 39; r_1_). Structure suggested admixed genetic patterns in the region in 56% (36/64) of all individuals, while DAPC assigned 77% with high posterior probability to one of the two clusters. According to the results of NewHybrids only eight individuals were assigned to parental group 1 (P1; purple) and one was determined to be a pure P2 (assignment probability >0.8). All remaining individuals in the population BB were designated hybrids.

#### Region SW (Fig. 8)

Mitochondrial haplotypes of the French (g) and the Scandinavian (f) relict populations were found in the Swiss region SW. DAPC and Structure analyses suggested the presence of two populations (*K* = 2) with seven individuals out of 32 showing an intermediate genotype in Structure. This admixture was confirmed by NewHybrids. This complies with a clear geographical distribution with Scandinavian haplotypes in the north-eastern and French haplotypes in western Switzerland.

#### Region GR

This region represented a special case in our study because of the co-occurrence of an additional beaver species (*C. canadensis*). All 20 identified *C. canadensis* showed an identical haplotype. Across *C. fiber*, however, this region harboured the highest number of haplotypes. Maternal lineages from French beavers (g, n = 4), Scandinavian beavers (f, n = 2), the potential Russian lineage (r_1_, n = 16), and the German relict haplotype (a_1_, n = 1) were found. Additionally, we found one individual bearing a mitochondrial haplotype (e) of the eastern ESU. Microsatellite analysis of the *C. fiber* samples revealed no clear population structure, with single samples that clustered with an admixed genotype in the same group as the southern German admixed population, the German relict population (*C. f. albicus*) or the eastern European beavers.

### Genetic Diversity

We found several significant deviations from HWE due to heterozygote deficits in all but the admixed BB population (Table 1, locus-specific information in Table S5). HWE departures disappeared in nearly all cases when single populations inferred from admixture analysis where used (exceptions were loci Cca18 and CF5 in the *C. f. albicus* group of Region EG and Cca8 in the *C. f. galliae* group of Region SW).

**Table 1:** Genetic diversity of North American and Eurasian beaver across the whole study, within the five study regions, and in the sub-populations within the study regions.

Region	sub-population	n	H_obs_±s.d.	H_exp_±s.d.	HWE dev.	A±s.d.	A_corr_±s.d.
***C. canadensis***	**–**	**20**	**0.49±0.30**	**0.52±0.20**	**2**	**2.35±0.88**	**N/A**
***C. fiber***	**–**	**215**	**0.35±0.07**	**0.61±0.12**	**N/A**	**4.85±1.63**	**4.14±1.16**
**Region HE**		**42**	**0.30±0.10**	**0.47±0.15**	**6**	**3.54±0.97**	**3.36±0.78**
	green	21	0.17±0.15	0.19±0.18	–	1.92±0.62	1.75±0.48
	red	14	0.49±0.13	0.54±0.11	–	3.08±0.86	3.00±0.73
**Region EG**		**53**	**0.28±0.11**	**0.40±0.12**	**4**	**3.62±1.03**	**3.33±1.00**
	green	30	0.18±0.26	0.22±0.21	–	1.85±0.86	1.65±0.69
	yellow	16	0.53±0.22	0.53±0.12	–	3.08±1.21	3.06±1.20
**Region BB**		**64**	**0.45±0.12**	**0.51±0.11**	**–**	**3.54±1.27**	**3.29±1.02**
	purple	13	0.45±0.25	0.41±0.19	–	2.38±0.74	2.37±0.72
	red	15	0.44±0.22	0.44±0.20	–	2.92±0.95	2.88±0.90
**Region SW**		**32**	**0.31±0.89**	**0.54±0.17**	**6**	**3.15±0.99**	**3.15±0.95**
	blue	12	0.49±0.15	0.50±0.10	–	2.46±0.84	2.45±0.83
	yellow	15	0.20±0.10	0.31±0.16	1	2.08±0.92	1.94±0.80
**Region GR**	**–**	**32**	**0.31±0.09**	**0.54±0.17**	**5**	**3.15±0.99**	**3.15±0.95**

**Figure 5:**
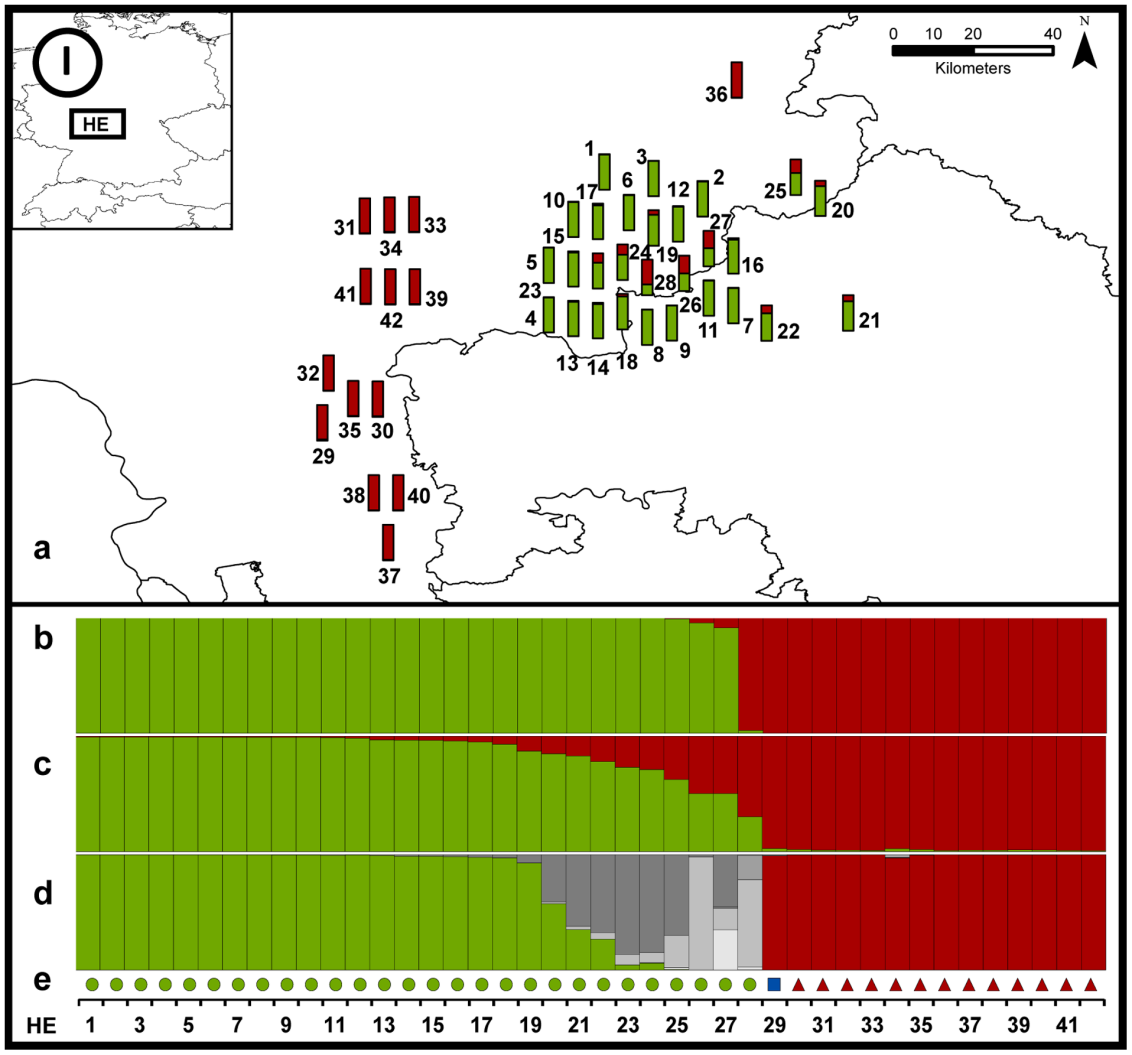
Detailed results of the genetic admixture for the study regions HE. Every bar symbolises one individual in the investigated areas (a). Colours indicate Structure population assignments. We also provide results of population assignments with DAPC (b), Structure (c) and NewHybrids (d). Shades of grey indicate assignment to one of the admixed hybrid classes, from light to dark grey: F_1_ hybrid, F_2_ hybrid, backcross to P_1_, and backcross to P_2_. Coloured dots show individual assignments to CR haplotypes (e) (green circle = *C. f. albicus* a_1_, blue square = *C. f. galliae* g, red triangle = *C. f. sp. r_1_*, yellow diamond = *C. f. fiber* f). For visual purposes samples found close to one another were slightly displaced. Exact coordinates and sample information can be found in Table S1.

See Fig. 5b–d for colour legend. Values are means with standard deviations (s.d.) across 13 microsatellite markers (11 for *C. canadensis*).

n = sample size.

H_obs_ = observed heterozygosity.

H_exp_ = expected heterozygosity.

HWE dev. = number of markers deviating from HWE.

A = observed allelic richness.

A_corr_ = allelic richness corrected for sample size for comparisons among and within regions.

s.d. = standard deviation.

Overall, allelic richness across loci was similarly moderate among regions, ranging from 3.15±0.95 to 4.14±1.16 (means of loci±s.d.). Single populations sorted according to Structure clustering revealed considerably lower values of genetic diversity, in particular in regions with pure occurrences of relict populations (e.g., *C. f. albicus* in EG) or its descendants (e.g., HE; Tab. 1). No significant differences of allelic richness between the two subgroups of each of the regions BB and SW (t-test; *p* = 0.14, both) were detectable whereas the allelic richness between the two subgroups within the regions HE and EG was significant different (*p*<0.001 and *p* = 0.002, respectively; Table S7).

## Discussion

By investigating 235 samples and a combination of mitochondrial and nuclear DNA markers we provide insight into hidden recolonisation and admixture processes over different temporal scales of admixture, from recent population contact such as observed for the SW or HE regions to the well-admixed beaver population in Southern Germany, where beavers of various population origins might already have started to admix since the 1960s.

### Mitochondrial DNA and Evolutionary Significant Units

The ESU is a fundamental concept in conservation biology. Moritz [12] defined ESUs as reciprocally monophyletic mtDNA units showing significant divergence of allele frequencies at nuclear markers. For reintroduction projects in particular, correctly choosing individuals for translocation is challenging. It is generally difficult to identify autochthonous populations fulfilling the ESU concept and often only small, potentially inbred relict populations are available. Eurasian beavers, for example, were extinct in most regions and relict populations were small and described as distinct subspecies based on morphology [8] and mitochondrial haplotypes [10]. The common classification into two ESUs corresponding to an eastern and a western lineage is popular and these two ESUs were also used as management units.

Except for one sample from GR, all analysed beaver individuals in our study bear western ESU haplotypes. This is surprising as reintroduction of beavers from eastern regions (Russia) to Central Europe was commonplace. Beavers reintroduced from Russia to Bavaria originate mainly from a Voronezh stock and were assumed to be of pure eastern ESU origin [13]. The lack of eastern haplotypes may be caused by Russian mtDNA lineages disappearing due to stochastic effects (lineage sorting). However, our reference samples from Kirov, Russia, also carry a western ESU haplotype and one of these haplotypes (r_1_) is the most common among our German samples. Furthermore it is the most similar haplotype (3 bp differences) compared to the described haplotype of the Elbe beaver (a_1_). The haplotype r_1_ was detected in HE, BB, EG and GR and we assume two different origins in Central Europe. For region BB we expect a direct reintroduction from Voronezh to Bavaria from where it spread also to region HE. For EG the haplotype, r_1_, appears near to the Polish border, so these animals may derive from Poland. Beavers with that haplotype were potentially bred in the beaver farm in Popielno, Poland, and built the source for reintroduction projects in Poland (also for reintroductions close to Germany). Given that many individuals from this farm came from Voronezh, Russia, [38] we can again conclude that the haplotype r_1_ is from Russia. Because of the reintroduction of beavers from the Popielno beaver farm and wild catches from Poland [39] to GR, we assume that beavers bearing the r_1_ haplotype derive from that farm.

The, so far, undescribed eastern lineage haplotype e was only found in GR. possibly implying that this lineage originated in Poland. Because we usually find only r_1_ haplotypes in regions where descendants from Popielno beavers live, we presume that the e haplotype stems from the wild population in Poland, potentially harbouring this eastern haplotype. It is likely, therefore, that a contemporary contact zone between the two ESUs appears in Poland.

The phylogenetic separation between the two ESUs has been dated to 210,000 years ago [40]. Due to the high potential of beavers to disperse along watersheds [8], [41] beavers started to recolonise huge areas and admixture between the separated mitochondrial lineages likely occurred. Consequently, the distribution of beavers was rather continuous from Europe to Asia for millennia while the two main lineages were still distinguishable, but the number of haplotypes was higher and the genetic distances between the “subspecies” lower [42]. Today’s observed mitochondrial differentiation of relict populations in “subspecies” is therefore an artifact of recent bottlenecks and the finding of three new *C. fiber* haplotypes within this study makes it likely that the general mitochondrial diversity is higher than expected. Further, ongoing natural dispersal and especially anthropogenic translocation increasingly leads to a breakdown of the historical, glaciation-induced, geographical separation of the major eastern and western lineages. The finding of western haplotypes in eastern regions (Kirov and Voronezh; Russia) suggests that the differentiation between the two ESUs is today not linked to geography as was previously suggested [10]. In Durka *et al.* [10] some of the samples of the relict populations were collected relatively far away from the original relict population areas, possibly explaining this discrepancy. We therefore suggest that individuals of both ESUs occur and perhaps also co-occur in some places in Eastern Eurasia.

The conclusions of other studies based on Halley’s [13] approach with the three schemes of beaver reintroductions (see Introduction) have to be revised based on the new results in this study and on the ancient DNA analysis of Horn *et al.* [42]. We suggest delineation of ESUs for *C. fiber* should in future be updated with nuclear markers once more samples from eastern regions are available. Nevertheless, we note that the analysis of mitochondrial control region sequences is valuable because it enables tracing the origin of beavers due to unique haplotypes in the extant European relict populations.

### Secondary Contact Zones and Subsequent Genetic Admixture

When generally comparing the different programs for admixture analysis, namely DAPC, Structure and NewHybrids, DAPC seemed over-confident when assigning individual cluster membership scores. Only a few individuals were identified as genetically admixed in DAPC as compared to Structure. Also NewHybrids suggested relatively high levels of genetic admixture (those identified as first, second, or later generation hybrids) in some regions. Methodologically, in DAPC there is a risk of over-fitting the discriminant functions when retaining too many principal components, which can lead to exaggerated and unstable posterior membership probabilities. The built-in DAPC function *optim.a.score* is supposed to balance discriminative power and over-fitting. However, while using this function as suggested, we suspected that for our data DAPC leads to results that under-estimate genetic admixture in the hybrid zones. Still, though to a lesser extent than Structure and NewHybrids, DAPC demonstrates the formation of hybrids zones where previously isolated beaver populations meet, and that these populations merge with on-going range expansion, most evident in BB. Additional evidence for higher extents of admixture as suggested by Structure and NewHybrids is the comparison with CR haplotype distribution. DAPC clustered one or several carriers of the CR haplotype of one population to the other population in HE, EG, and SW. NewHybrids identified all these unclear assignments as hybrids (e.g. individuals HE28, SW18, 19; Fig. 5 and Fig. 8).

**Figure 6:**
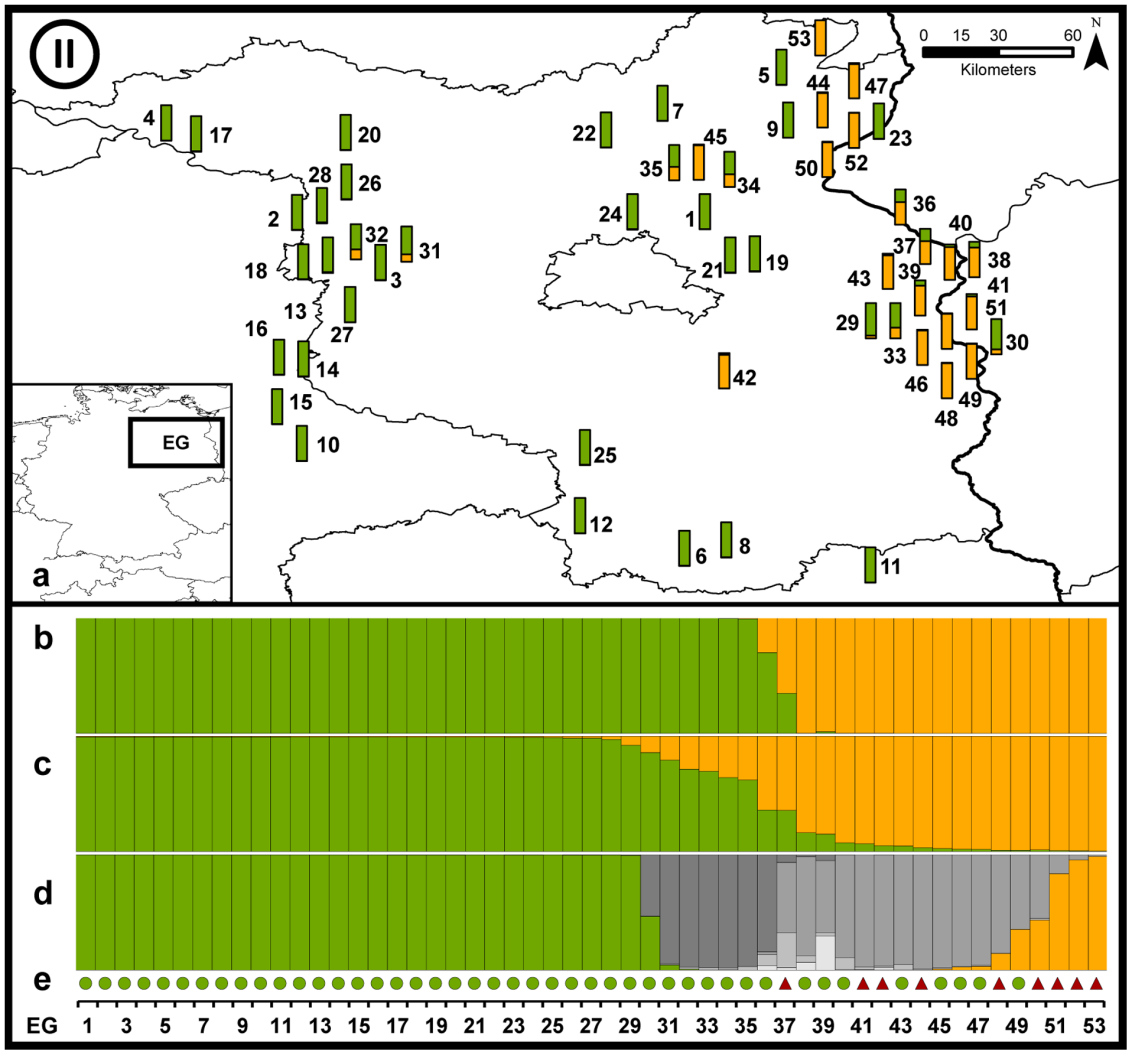
Detailed results of the genetic admixture for region EG. See Fig. 5 for details.

**Figure 7:**
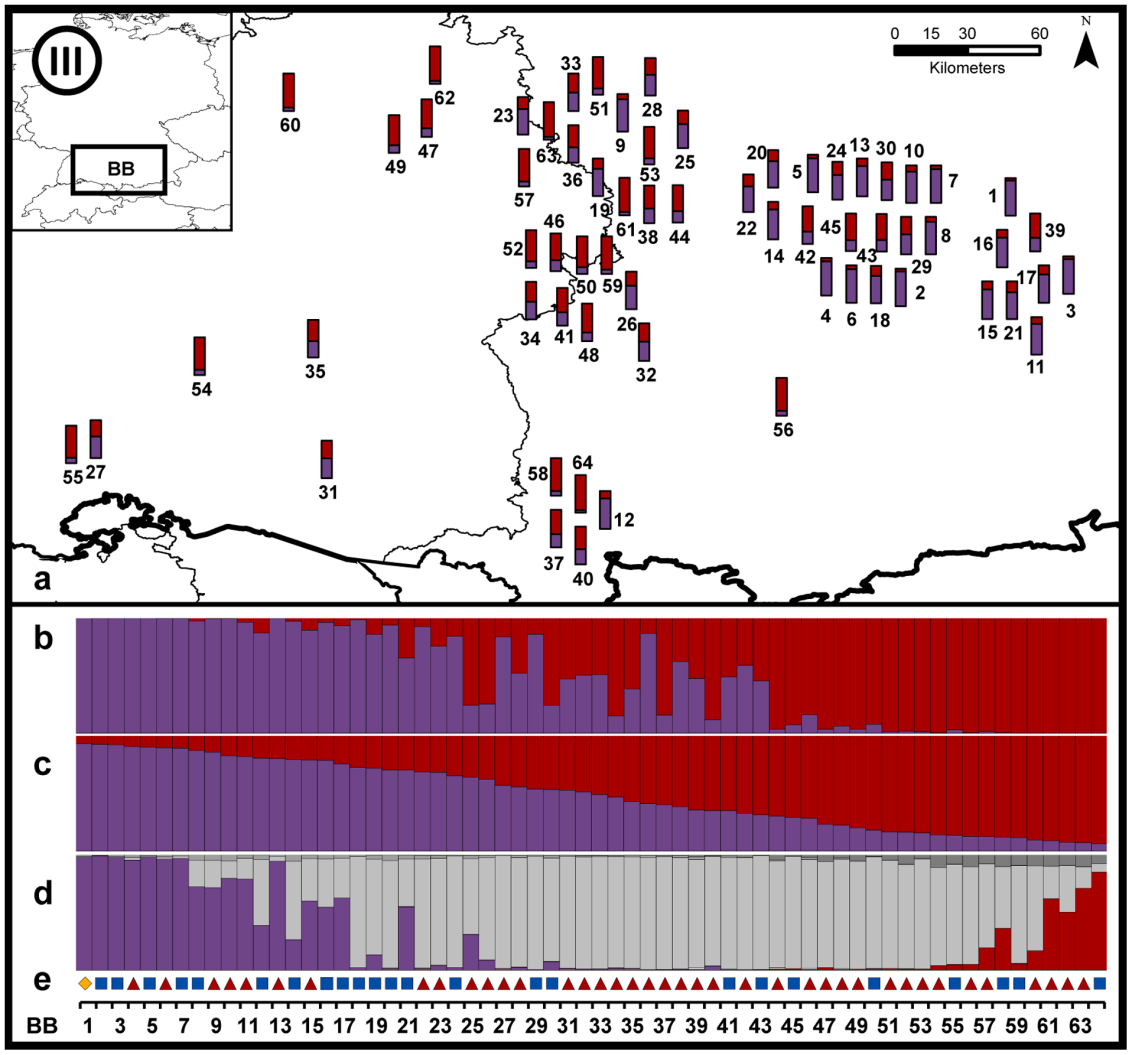
Detailed results of the genetic admixture for region BB. See Fig. 5 for details.

**Figure 8:**
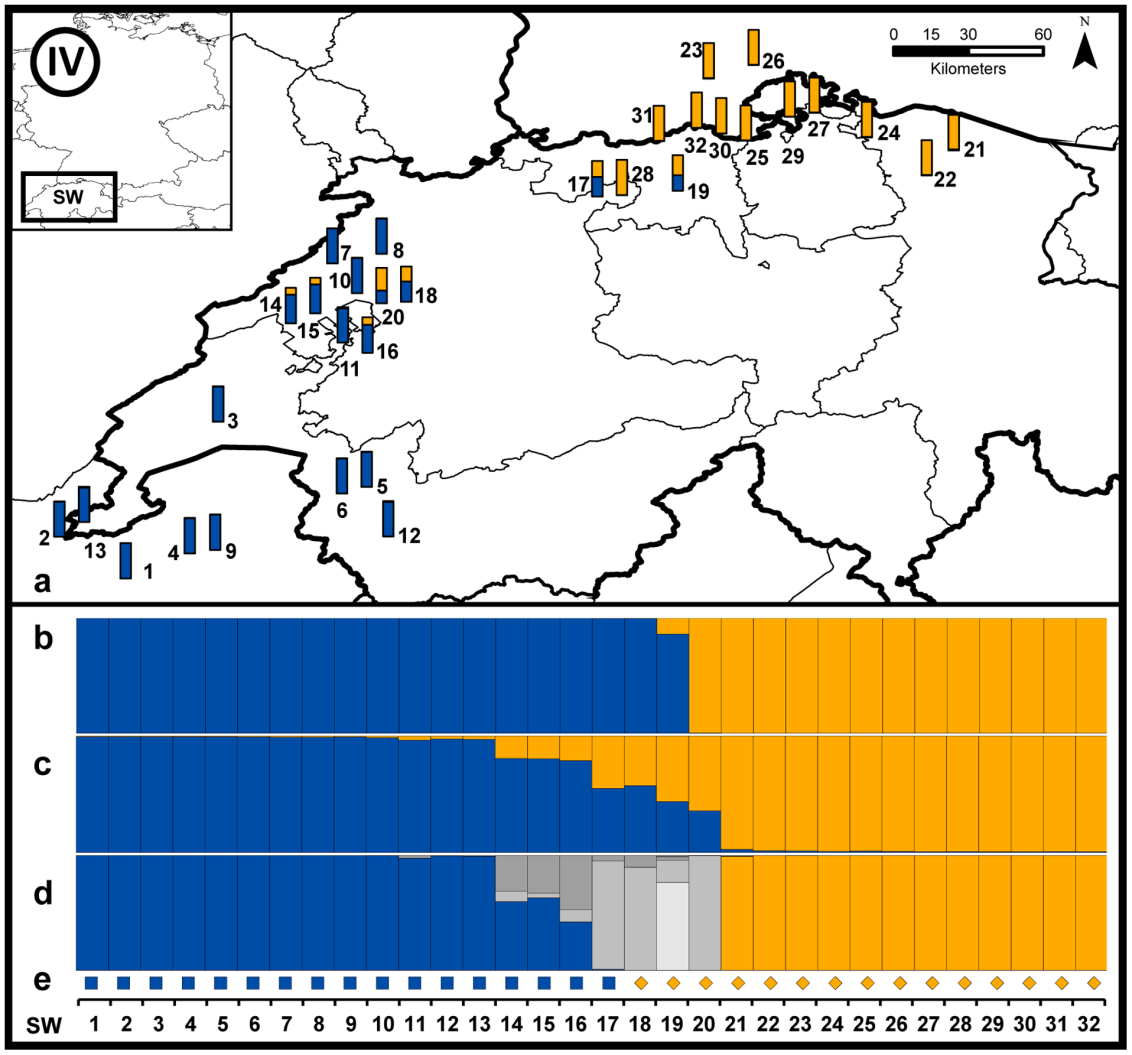
Detailed results of the genetic admixture for region SW. See Fig. 5 for details.

Whereas BB seemed nearly homogeneously admixed, we found differing degrees of genetic introgression within regions HE, SW and EG. For HE we explicitly showed that the current increase of the beaver population is not exclusively due to the successful dispersion of the reintroduced population in Hesse but due to the fast expansion of the bordering admixed population BB within the last 45 years. In BB, it is still possible to genetically identify originally reintroduced *C. f. albicus* and immigrants, respectively, while incipient admixture of the gene pool is detectable - also in the core zone of the reintroduced population: Sample HE26 from 2009 shows an admixed genotype indicating that admixture upon population contact has commenced. In SW we found incipient admixture between reintroduced French individuals and the reintroduced Scandinavian beavers in the Rhine river system while the population in the Rhône river system was still of pure French origin. Individuals from EG showed differing assignments in DAPC and Structure whereas NewHybrids indicated progressive admixture of the two populations. Nevertheless, a distinction of two populations in EG is still possible and especially the individuals of the western part of EG are clearly separated, indicating that autochthonous relict populations of *C. f. albicus* still persist without major introgression from other beaver lineages.

This incomplete admixture becomes evident when we consider that the sub-populations for HE, SW and EG were in HWE whereas the analysis of all beavers from the particular regions showed strong deviations. These deviations in the “combined” region carried the typical signature of a Wahlund [43] effect, i.e., heterozygote deficit due to non-random mating in sub-structured populations. In contrast, the genetic uniformity of BB resulted in no region-wide deviations from HWE.

The different degrees of admixture also become apparent when considering the distribution of the maternal lineage. The fact that all investigated beavers from the Spessart area (HE) still show the a_1_ haplotype indicates that beaver dispersal in this region is male-triggered. Conversely, females from Poland contributed to the recolonisation process in the contact zone EG as evidenced by the local occurrence of both haplotypes. Similarly, in SW individuals SW17-19 carry a haplotype not matching their locality in the geographically separated groups of reintroduced French and Scandinavian beavers. Interestingly, no haplotype from reintroduced Russian beavers was found in SW even though multiple reintroductions from Russian stock were performed. However, the clustering analyses of the entire data set assigned the population from eastern EG (including the immigrated individuals from Poland with Russian ancestry) and northern Switzerland (yellow; Fig. 3; Fig. 8) to one cluster. Thus, we conclude that nuclear introgression of reintroduced Russian beavers took place in the Swiss population and that we either missed samples with a maternal lineage of Russian origin or this might be directional hybridization.

### Inbreeding *vs.* Outbreeding

Only few beaver individuals survived human persecution in the known beaver relict populations throughout Eurasia [44], suggesting strong bottlenecks, inbreeding, and reduced adaptive potential. Nolet & Rosell [7] estimated a minimum viable census population size for *C. fiber* to be 1,880, a value well above the population census in all relict populations. Human-mediated reintroductions of few individuals from already bottlenecked populations to other areas resulted in even more severe bottlenecks and the low genetic diversity generally found in beaver populations today [9].

A low population growth rate was documented for a Dutch population where 42 *C. f. albicus* from the German Elbe river relict population were reintroduced [41]. Similarly, the Hessian population originating from 18 *C. f. albicus* individuals still forms a population of less than 1,000 individuals. This population remained in the introduction region for ∼25 years, and have the lowest heterozygosity values in this study. On the other extreme, the Bavarian population, founded by around 43 individuals from different relict populations rose to a population of ∼14,000 individuals, which then actively recolonised adjacent areas over a 45 year period. Note, however, that the comparison of these populations is difficult because the population growth could be exponential, something we cannot see with our data. These observations provide evidence for the hypothesis that population growth rates and dispersal might be governed by the level of genetic diversity and inbreeding. Further research integrating ecological and demographic data will aid investigating this idea.

It has been hypothesised that beaver populations might carry low rates of genetic load because of a lack of observed inbreeding depression, even in small and bottlenecked populations [9], [44]. However, evidence for inbreeding depression at least in the *C. f. albicus* population, which has been used for multiple reintroduction projects, has been observed in small beaver populations (e.g. small litter size [6], high susceptibility to epidemic diseases [9], [39], and jaw abnormalities [45]). The low heterozygosity in the native and the reintroduced *C. f. albicus* population suggests that high genetic load is a plausible threat to *C. f. albicus*.

Although experiments are required to test for outbreeding depression, we have no empirical evidence for its effects in this study. Beavers of different relict populations from the western ESU have merged successfully and formed stable populations. Haplotype analysis detected most or all source populations used in the reintroductions. Findings from Russia, where admixed populations show higher reproductive rates and are more resilient to hunting pressure [46] support our tentative assumptions that potential outbreeding depression is of minor importance in beavers.

### Management Implications

In contrast to previous assumptions made by Dewas *et al.* [16] and references therein, we did not detect any *C. canadensis* in most regions, including the highly admixed Southern German population. As this population has served as source population for numerous reintroduction programs [47], this finding is of importance for beaver managers across Europe [48]. However, we confirmed the presence of *Cc* in the Greater Region. This species was first detected in GR in 2006, likely as the result of zoo escapees [16]. We recommend eradication before they potentially spread as was the case in Finland. There, seven North American beavers were introduced in 1937 and the population subsequently increased to 12,000 individuals over the following 64 years [49]. Current eradication and sterilisation programs assisted by genetic species assignment are ongoing in GR in order to halt the potential spread of the species in Central Europe.

There is a continuing discussion among beaver managers and stakeholders in reintroduction areas about the appropriateness of beaver reintroductions from different origins, for instance using admixed stocks from Bavaria e.g., [16]. The populations in our study, including those from Bavaria (Region BB) nearly exclusively consist of beavers carrying a western mitochondrial haplotype. Horn *et al*. [42] show that beavers in Western Europe, including the study region, consisted of western haplotypes but historical haplotype variation was much greater than today and the described “subspecies” are an artefact of recent anthropogenic bottlenecks. This leads to the conclusion that using admixed beavers from any of the western ESUs as source population for reintroduction programs in Western Europe is well justified. While we do not want to interfere with ongoing conservation planning aimed at protection the purebred relict populations in Europe [50], e.g. *C. f. albicus* in Germany, we at least question the long-term appropriateness of this approach. All analysed beaver populations originating from the Elbe relict population are severely impoverished at most or all analysed loci, indicating strong historic bottleneck effects and inbreeding. Diversity values increase considerably when two populations get admixed as we found in EG or HE, and are highest in regions where individuals from several relict populations were reintroduced. While further studies on the effects of inbreeding and outbreeding on the fitness of beaver populations are clearly needed, the fact that outcrossed populations are thriving in several regions suggests that so far there is no reason to prevent population admixture and increase of genetic diversity in local beaver populations. It also has to remain open if the conservation of purebred lineages, such as *C. f. albicus* would be feasible from a practical point of view. As admixture is ongoing in all of our study regions, the maintenance of purebred lineages would require extensive genetic sampling, along with strict relocation or eradication programs for introduced beaver lineages. Such procedure seems unachievable given the steadily increasing number of beavers in Germany and neighboring regions. Moreover, based on the existing genetic data we strongly question the appropriateness of such action. However, we cannot exclude the possibility of potential future disappearance of pure relict lineages, such as *C.f. albicus*, due to ongoing admixture.

To solve the existing phylogeographic uncertainties in *C. fiber* and the geographic distribution of its haplotypes and ESUs further analyses of both mitochondrial and nuclear DNA from samples from Eastern Europe and Russia are required. For this, we recommend combining our approach with additional nuclear marker sets, such as recently developed SNP panels [51]. These genome-wide marker systems will also help to study the potential effects of inbreeding and outbreeding in beaver populations and will aid its conservation and population management.

## Supporting Information

Table S1 **Detailed samples information.**
(DOCX)

Table S2 **Overview of successfully analysed samples for the five regions.**
(DOCX)

Table S3 **Error rates of the 57 analysed hair samples.**
(DOCX)

Table S4 **Microsatellites evaluated and used in this study.**
(DOCX)

Table S5 **Observed and expected heterozygosity and deviations from Hardy-Weinberg equilibrium per region and per locus.**
(DOCX)

Table S6 **Overview of haplotypes.**
(DOCX)

Table S7 **Allelic richness across regions.**
(DOCX)

Table S8 **Genotype data.**
(TXT)

Text S1 **Additional text describing details regarding the beaver population history in each region.**
(DOCX)
