# Supplementary material for: The Genetic Legacy of Multiple Beaver Reintroductions in Central Europe
Source: PLoS One. 2014 May 14;9(5):e97619. doi: 10.1371/journal.pone.0097619 (PMC4020922; doi:10.1371/journal.pone.0097619)
Supplement: Table S1 — Detailed samples information. (DOCX) [file pone.0097619.s001.docx]

**Supplementary Table S1** Detailed sample information. Sample ID including region information, internal sample ID, sample type (t = tissue, h = hair; subscript indicates means of sample acquisition), CR-haplotypes (a_1_ = *C. f. albicus* DQ088700, g = *C. f. galliae* DQ088703*,* r_1_ = *C. f. sp.* JF264887, f *= C. f. fiber* DQ088702, e = KF731637, c = *C. canadensis* KF731638), decimal GPS-coordinates, sampling date and collecting person.

| Region & Sample ID | Internal sample ID | Sample type | | CR | x-coordinates | y-coordinates | Sampling date | Collected by |
| --- | --- | --- | --- | --- | --- | --- | --- | --- |
| HE01 | CA042 | t_FD_ | a_1_ | | 9.536 | 50.262 | 31.03.2010 | E & J Schultheis |
| HE02 | CA003 | t_FD_ | a_1_ | | 9.672 | 50.262 | 12.12.2008 | F Müller |
| HE03 | CA034 | t_FD_ | a_1_ | | 9.528 | 50.263 | 13.08.2008 | F Müller |
| HE04 | CA057 | t_FD_ | a_1_ | | 9.482 | 50.240 | 31.12.2010 | P Könnemann |
| HE05 | CB001 | t_FD_ | a_1_ | | 9.510 | 50.256 | 06.01.2011 | P Könnemann |
| HE06 | CC114 | t_FD_ | a_1_ | | 9.526 | 50.345 | 01.03.2012 | F Müller |
| HE07 | CC113 | t_FD_ | a_1_ | | 9.668 | 50.258 | 13.03.2012 | F Müller |
| HE08 | CB039 | h_NS_ | a_1_ | | 9.475 | 50.242 | 17.04.2011 | U Beschke |
| HE09 | CB028 | h_NS_ | a_1_ | | 9.589 | 50.267 | 23.04.2011 | R Loos |
| HE10 | CB085 | h_NS_ | a_1_ | | 9.455 | 50.244 | 03.07.2011 | R Ruppel |
| HE11 | CB033 | h_NS_ | a_1_ | | 9.619 | 50.261 | 17.04.2011 | R Loos |
| HE12 | CC111 | t_FD_ | a_1_ | | 9.633 | 50.267 | 06.04.2012 | F Müller |
| HE13 | CB040 | h_NS_ | a_1_ | | 9.476 | 50.242 | 12.04.2011 | U Beschke |
| HE14 | CC019 | t_FD_ | a_1_ | | 9.505 | 50.255 | 15.03.2012 | F Müller |
| HE15 | CB071 | h_NS_ | a_1_ | | 9.483 | 50.247 | 07.04.2011 | E & J Schultheis |
| HE16 | CA033 | t_FD_ | a_1_ | | 9.709 | 50.277 | 11.01.2007 | F Müller |
| HE17 | CB073 | h_NS_ | a_1_ | | 9.541 | 50.262 | 12.04.2011 | E & J Schultheis |
| HE18 | CB070 | h_NS_ | a_1_ | | 9.538 | 50.262 | 06.04.2011 | E & J Schultheis |
| HE19 | CB096 | t_FD_ | a_1_ | | 9.515 | 50.258 | 23.10.2011 | F Müller |
| HE20 | CC109 | t_FD_ | a_1_ | | 9.953 | 50.406 | 08.05.2012 | F Müller |
| HE21 | CA045 | t_FD_ | a_1_ | | 10.021 | 50.148 | 01.03.2010 | F Müller |
| HE22 | CC110 | t_FD_ | a_1_ | | 9.837 | 50.125 | 10.05.2012 | F Müller |
| HE23 | CB046 | h_NS_ | a_1_ | | 9.476 | 50.243 | 14.04.2011 | U Beschke |
| HE24 | CB078 | h_NS_ | a_1_ | | 9.529 | 50.263 | 06.04.2011 | E & J Schultheis |
| HE25 | CA053 | t_FD_ | a_1_ | | 9.902 | 50.454 | 05.10.2010 | F Müller |
| HE26 | CA044 | t_FD_ | a_1_ | | 9.674 | 50.237 | 20.06.2009 | F Müller |
| HE27 | CB095 | t_FD_ | a_1_ | | 9.706 | 50.293 | 02.10.2011 | F Müller |
| HE28 | CA004 | t_FD_ | a_1_ | | 9.567 | 50.228 | 01.03.2010 | P Könnemann |
| HE29 | CC183 | t_FD_ | g | | 8.835 | 49.901 | 13.05.2012 | J Siek |
| HE30 | CC170 | h_NS_ | r_1_ | | 8.906 | 49.954 | 17.04.2012 | L Seipp |
| HE31 | CC140 | h_NS_ | r_1_ | | 8.983 | 50.370 | 10.05.2012 | T Allert |
| HE32 | CC177 | h_NS_ | r_1_ | | 8.903 | 49.952 | 21.04.2012 | L Seipp |
| HE33 | CC135 | h_NS_ | r_1_ | | 8.983 | 50.367 | 28.04.2012 | T Allert |
| HE34 | CC139 | h_NS_ | r_1_ | | 8.986 | 50.369 | 29.04.2012 | T Allert |
| HE35 | CC166 | h_NS_ | r_1_ | | 8.904 | 49.955 | 22.04.2012 | L Seipp |
| HE36 | CC011 | t_FD_ | r_1_ | | 9.769 | 50.673 | 18.12.2011 | F Müller |
| HE37 | CC123 | h_NS_ | r_1_ | | 8.983 | 49.673 | 21.04.2012 | K Rapp |
| HE38 | CC127 | h_NS_ | r_1_ | | 8.988 | 49.730 | 15.04.2012 | K Rapp |
| HE39 | CC150 | h_NS_ | r_1_ | | 8.986 | 50.207 | 22.04.2012 | M Sattler |
| HE40 | CC121 | h_NS_ | r_1_ | | 9.006 | 49.743 | 18.04.2012 | K Rapp |
| HE41 | CC147 | h_NS_ | r_1_ | | 8.985 | 50.208 | 18.04.2012 | M Sattler |
| HE42 | CC152 | h_NS_ | r_1_ | | 8.988 | 50.207 | 14.04.2012 | M Sattler |
| EG01 | CB116 | t_FD_ | a_1_ | | 13.722 | 52.720 | 20.04.2005 | J & J Teubner |
| EG02 | CB113 | t_FD_ | a_1_ | | 12.269 | 52.677 | 04.04.2005 | J & J Teubner |
| EG03 | CB125 | t_FD_ | a_1_ | | 12.467 | 52.500 | 04.08.2008 | J & J Teubner |
| EG04 | CB128 | t_FD_ | a_1_ | | 11.747 | 53.004 | 12.04.2008 | J & J Teubner |
| EG05 | CB136 | t_FD_ | a_1_ | | 13.972 | 53.206 | 21.05.2007 | J & J Teubner |
| EG06 | CB139 | t_FD_ | a_1_ | | 13.621 | 51.468 | 18.12.2003 | J & J Teubner |
| EG07 | CB140 | t_FD_ | a_1_ | | 13.542 | 53.075 | 05.04.2005 | J & J Teubner |
| EG08 | CB143 | t_FD_ | a_1_ | | 13.773 | 51.498 | 07.05.2004 | J & J Teubner |
| EG09 | CB144 | t_FD_ | a_1_ | | 13.997 | 53.015 | 23.02.2005 | J & J Teubner |
| EG10 | CC018 | t_FD_ | a_1_ | | 12.238 | 51.846 | 29.05.1981 | U Müller & M Krüger |
| EG11 | CC022 | t_FD_ | a_1_ | | 14.294 | 51.398 | 31.08.2010 | V Harms |
| EG12 | CB104 | t_FD_ | a_1_ | | 13.244 | 51.585 | 30.11.2006 | J & J Teubner |
| EG13 | CB138 | t_FD_ | a_1_ | | 12.412 | 52.407 | 25.06.2004 | J & J Teubner |
| EG14 | CC057 | h_FD_ | a_1_ | | 12.212 | 52.151 | 2009-2012 | J Michaux |
| EG15 | CC058 | h_FD_ | a_1_ | | 12.172 | 52.072 | 2009-2012 | J Michaux |
| EG16 | CC056 | h_FD_ | a_1_ | | 12.155 | 52.158 | 2009-2012 | J Michaux |
| EG17 | CB106 | t_FD_ | a_1_ | | 11.856 | 52.965 | 10.09.2008 | J & J Teubner |
| EG18 | CB124 | t_FD_ | a_1_ | | 12.331 | 52.503 | 06.10.2004 | J & J Teubner |
| EG19 | CB135 | t_FD_ | a_1_ | | 13.821 | 52.531 | 27.05.2002 | J & J Teubner |
| EG20 | CB146 | t_FD_ | a_1_ | | 12.396 | 52.943 | 07.09.2009 | J & J Teubner |
| EG21 | CB100 | t_FD_ | a_1_ | | 13.787 | 52.527 | 18.02.2010 | J & J Teubner |
| EG22 | CB133 | t_FD_ | a_1_ | | 13.338 | 52.979 | 02.05.2006 | J & J Teubner |
| EG23 | CB105 | t_FD_ | a_1_ | | 14.222 | 53.010 | 02.12.2008 | J & J Teubner |
| EG24 | CB117 | t_FD_ | a_1_ | | 13.433 | 52.683 | 21.04.2004 | J & J Teubner |
| EG25 | CB121 | t_FD_ | a_1_ | | 13.261 | 51.832 | 28.05.2008 | J & J Teubner |
| EG26 | CB127 | t_FD_ | a_1_ | | 12.370 | 52.791 | 04.11.2009 | J & J Teubner |
| EG27 | CB134 | t_FD_ | a_1_ | | 12.332 | 52.561 | 02.03.2008 | J & J Teubner |
| EG28 | CB122 | t_FD_ | a_1_ | | 12.310 | 52.705 | 08.09.2003 | J & J Teubner |
| EG29 | CB120 | t_FD_ | a_1_ | | 14.395 | 52.399 | 08.04.2005 | J & J Teubner |
| EG30 | CB142 | t_FD_ | a_1_ | | 14.588 | 52.232 | 28.03.2004 | J & J Teubner |
| EG31 | CB109 | t_FD_ | a_1_ | | 12.617 | 52.567 | 04.06.2011 | J & J Teubner |
| EG32 | CB103 | t_FD_ | a_1_ | | 12.433 | 52.575 | 19.09.2008 | J & J Teubner |
| EG33 | CB114 | t_FD_ | a_1_ | | 14.533 | 52.343 | 12.11.2001 | J & J Teubner |
| EG34 | CB123 | t_FD_ | a_1_ | | 13.785 | 52.837 | 20.05.2007 | J & J Teubner |
| EG35 | CB098 | t_FD_ | a_1_ | | 13.691 | 52.861 | 07.05.2007 | J & J Teubner |
| EG36 | CB107 | t_FD_ | a_1_ | | 14.402 | 52.700 | 27.10.2008 | J & J Teubner |
| EG37 | CB118 | t_FD_ | r_1_ | | 14.550 | 52.559 | 08.04.2002 | J & J Teubner |
| EG38 | CB130 | t_FD_ | a_1_ | | 14.598 | 52.511 | 26.01.2003 | J & J Teubner |
| EG39 | CB102 | t_FD_ | a_1_ | | 14.473 | 52.372 | 05.05.2010 | J & J Teubner |
| EG40 | CB101 | t_FD_ | a_1_ | | 14.581 | 52.502 | 29.09.2008 | J & J Teubner |
| EG41 | CB141 | t_FD_ | r_1_ | | 14.536 | 52.237 | 14.10.2003 | J & J Teubner |
| EG42 | CB129 | t_FD_ | r_1_ | | 13.765 | 52.109 | 09.04.2006 | J & J Teubner |
| EG43 | CB115 | t_FD_ | a_1_ | | 14.357 | 52.468 | 01.05.2005 | J & J Teubner |
| EG44 | CB111 | t_FD_ | r_1_ | | 14.235 | 53.039 | 26.03.2007 | J & J Teubner |
| EG45 | CB097 | t_FD_ | a_1_ | | 13.673 | 52.863 | 01.04.2008 | J & J Teubner |
| EG46 | CB119 | t_FD_ | a_1_ | | 14.537 | 52.265 | 08.03.2004 | J & J Teubner |
| EG47 | CB132 | t_FD_ | a_1_ | | 14.121 | 52.976 | 18.03.2003 | J & J Teubner |
| EG48 | CB126 | t_FD_ | r_1_ | | 14.646 | 52.089 | 30.09.2009 | J & J Teubner |
| EG49 | CB108 | t_FD_ | a_1_ | | 14.642 | 52.144 | 22.01.2007 | J & J Teubner |
| EG50 | CB099 | t_FD_ | r_1_ | | 14.138 | 52.873 | 09.04.2010 | J & J Teubner |
| EG51 | CB131 | t_FD_ | r_1_ | | 14.571 | 52.253 | 15.12.2004 | J & J Teubner |
| EG52 | CB137 | t_FD_ | r_1_ | | 14.235 | 52.978 | 21.04.2007 | J & J Teubner |
| EG53 | CB145 | t_FD_ | r_1_ | | 12.471 | 52.280 | 18.06.2007 | J & J Teubner |
| BB01 | CA035 | t_FD_ | f | | 12.027 | 48.721 | 21.02.2010 | B-J Haag |
| BB02 | CA041 | t_FD_ | g | | 11.485 | 48.766 | 31.03.2010 | R Zange |
| BB03 | CB003 | t_FD_ | g | | 12.108 | 48.559 | 07.02.2011 | B-J Haag |
| BB04 | CA054 | t_FD_ | r_1_ | | 11.349 | 48.796 | 26.11.2010 | R Zange |
| BB05 | CA023 | t_C_ | g | | 11.420 | 48.787 | 05.01.2010 | R Zange |
| BB06 | CA056 | t_FD_ | r_1_ | | 11.399 | 48.740 | 03.12.2010 | R Zange |
| BB07 | CA039 | t_C_ | g | | 11.487 | 48.768 | 26.03.2010 | R Zange |
| BB08 | CA040 | t_C_ | g | | 11.489 | 48.767 | 28.03.2010 | R Zange |
| BB09 | CA012 | t_FD_ | r_1_ | | 10.484 | 49.163 | 30.11.2009 | G Engelhard |
| BB10 | CB002 | t_FD_ | r_1_ | | 11.451 | 48.784 | 28.01.2011 | R Zange |
| BB11 | CA026 | t_C_ | r_1_ | | 11.945 | 48.463 | 01.01.2010 | G Schwab |
| BB12 | CA052 | t_FD_ | g | | 10.336 | 47.685 | 01.06.2010 | W Adam |
| BB13 | CA024 | t_C_ | r_1_ | | 11.391 | 48.923 | 01.01.2010 | G Schwab |
| BB14 | CA028 | t_C_ | g | | 11.033 | 48.762 | 01.01.2010 | G Schwab |
| BB15 | CA025 | t_C_ | r_1_ | | 11.948 | 48.469 | 01.01.2010 | G Schwab |
| BB16 | CA032 | t_FD_ | g | | 11.912 | 48.638 | 03.02.2010 | B-J Haag |
| BB17 | CA036 | t_FD_ | g | | 12.068 | 48.583 | 14.03.2010 | B-J Haag |
| BB18 | CB169 | t_FD_ | g | | 11.443 | 48.651 | 06.12.2011 | R Zange |
| BB19 | CA047 | t_FD_ | g | | 10.407 | 48.923 | 03.10.2010 | R Allgöwer |
| BB20 | CA018 | t_C_ | g | | 11.008 | 48.870 | 01.12.2009 | G Schwab |
| BB21 | CA027 | t_C_ | g | | 11.949 | 48.466 | 01.01.2010 | G Schwab |
| BB22 | CA017 | t_C_ | r_1_ | | 10.968 | 48.863 | 01.12.2009 | G Schwab |
| BB23 | CB016 | t_FD_ | r_1_ | | 10.129 | 49.152 | 22.03.2011 | R Allgöwer |
| BB24 | CB006 | t_FD_ | g | | 11.347 | 48.791 | 04.03.2011 | R Zange |
| BB25 | CA009 | t_FD_ | r_1_ | | 10.724 | 49.102 | 01.12.2009 | J Schlüter |
| BB26 | CC112 | t_FD_ | r_1_ | | 10.509 | 48.499 | 01.04.2012 | F Müller |
| BB27 | CC073 | t_FD_ | r_1_ | | 8.530 | 47.948 | 14.04.2012 | B Sättele |
| BB28 | CA005 | t_FD_ | r_1_ | | 10.602 | 49.297 | 19.11.2009 | W Kieslinger |
| BB29 | CA043 | t_FD_ | g | | 11.438 | 48.706 | 13.04.2010 | R Zange |
| BB30 | CB005 | t_FD_ | g | | 11.425 | 48.762 | 25.02.2011 | R Zange |
| BB31 | CA013 | t_FD_ | r_1_ | | 9.397 | 47.873 | 04.07.2009 | R Allgöwer |
| BB32 | CA010 | t_FD_ | r_1_ | | 10.579 | 48.310 | 28.11.2009 | J Schmid |
| BB33 | CB017 | t_FD_ | r_1_ | | 10.234 | 49.155 | 22.03.2011 | R Allgöwer |
| BB34 | CA051 | t_FD_ | r_1_ | | 10.209 | 48.644 | 01.06.2010 | R Allgöwer |
| BB35 | CA048 | t_FD_ | r_1_ | | 9.347 | 48.323 | 03.05.2010 | R Allgöwer |
| BB36 | CA046 | t_FD_ | r_1_ | | 10.291 | 49.047 | 13.08.2009 | R Allgöwer |
| BB37 | CB092 | t_FD_ | r_1_ | | 10.292 | 47.733 | 19.08.2011 | W Adam |
| BB38 | CA030 | t_C_ | r_1_ | | 10.516 | 48.823 | 01.12.2009 | G Schwab |
| BB39 | CA038 | t_FD_ | r_1_ | | 12.036 | 48.718 | 04.03.2010 | B-J Haag |
| BB40 | CB013 | t_FD_ | r_1_ | | 10.317 | 47.704 | 15.03.2011 | W Adam |
| BB41 | CB167 | t_FD_ | g | | 10.278 | 48.452 | 29.11.2011 | F Kopp |
| BB42 | CA029 | t_FD_ | r_1_ | | 11.188 | 48.745 | 01.12.2009 | G Schwab |
| BB43 | CB004 | t_FD_ | g | | 11.463 | 48.716 | 13.02.2011 | R Zange |
| BB44 | CA016 | t_C_ | r_1_ | | 10.705 | 48.824 | 01.12.2009 | G Schwab |
| BB45 | CB168 | t_FD_ | g | | 11.349 | 48.718 | 05.12.2011 | R Zange |
| BB46 | CA014 | t_FD_ | r_1_ | | 10.209 | 48.644 | 01.05.2007 | R Allgöwer |
| BB47 | CB018 | t_FD_ | r_1_ | | 9.769 | 49.143 | 10.01.2011 | R Allgöwer |
| BB48 | CA006 | t_FD_ | r_1_ | | 10.367 | 48.383 | 20.11.2009 | O Frimmel |
| BB49 | CB015 | t_FD_ | r_1_ | | 9.648 | 49.084 | 10.12.2010 | R Allgöwer |
| BB50 | CA019 | t_FD_ | g | | 10.426 | 48.633 | 02.04.2009 | R Allgöwer |
| BB51 | CA007 | t_FD_ | r_1_ | | 10.407 | 49.300 | 21.11.2009 | W Kieslinger |
| BB52 | CA021 | t_FD_ | r_1_ | | 10.158 | 48.657 | 18.03.2009 | R Allgöwer |
| BB53 | CA008 | t_FD_ | r_1_ | | 10.597 | 49.041 | 23.11.2009 | G Engelhard |
| BB54 | CC072 | t_FD_ | r_1_ | | 8.924 | 48.257 | 13.04.2012 | B Sättele |
| BB55 | CC001 | h_FD_ | g | | 8.445 | 47.929 | 13.12.2011 | B Sättele |
| BB56 | CA037 | t_FD_ | r_1_ | | 11.091 | 48.106 | 15.03.2004 | B-J Haag |
| BB57 | CA050 | t_FD_ | r_1_ | | 10.195 | 49.126 | 19.04.2010 | R Allgöwer |
| BB58 | CC118 | t_FD_ | g | | 10.289 | 47.808 | 25.04.2012 | W Adam |
| BB59 | CA020 | t_FD_ | g | | 10.440 | 48.633 | 30.03.2009 | R Allgöwer |
| BB60 | CB019 | t_FD_ | r_1_ | | 9.255 | 49.240 | 29.03.2011 | R Allgöwer |
| BB61 | CA015 | t_C_ | r_1_ | | 10.506 | 48.851 | 01.12.2009 | G Schwab |
| BB62 | CB020 | t_FD_ | r_1_ | | 9.801 | 49.341 | 14.04.2011 | R Allgöwer |
| BB63 | CA049 | t_FD_ | r_1_ | | 10.224 | 49.133 | 19.04.2010 | R Allgöwer |
| BB64 | CB014 | t_FD_ | g | | 10.343 | 47.745 | 04.04.2011 | W Adam |
| SW01 | CC066 | t_FD_ | g | | 6.221 | 45.776 | 20.11.2009 | J Michaux |
| SW02 | CC078 | t_FD_ | g | | 5.968 | 46.138 | 26.04.2007 | C Angst |
| SW03 | CC085 | t_FD_ | g | | 6.511 | 46.552 | 08.08.2007 | C Angst |
| SW04 | CC065 | t_FD_ | g | | 6.409 | 46.078 | 05.06.2009 | J Michaux |
| SW05 | CC079 | t_FD_ | g | | 6.961 | 46.270 | 14.05.2007 | C Angst |
| SW06 | CC082 | t_FD_ | g | | 6.958 | 46.293 | 20.07.2007 | C Angst |
| SW07 | CC088 | t_FD_ | g | | 7.034 | 47.016 | 15.04.2008 | C Angst |
| SW08 | CC097 | t_FD_ | g | | 7.059 | 47.041 | 11.01.2009 | C Angst |
| SW09 | CC064 | t_FD_ | g | | 6.501 | 46.091 | 29.112.009 | J Michaux |
| SW10 | CC096 | t_FD_ | g | | 7.046 | 47.016 | 19.10.2009 | C Angst |
| SW11 | CC081 | t_FD_ | g | | 7.025 | 46.915 | 25.05.2007 | C Angst |
| SW12 | CC074 | t_FD_ | g | | 7.125 | 46.139 | 13.01.1996 | C Angst |
| SW13 | CC080 | t_FD_ | g | | 6.028 | 46.191 | 04.06.2007 | C Angst |
| SW14 | CC100 | t_FD_ | g | | 6.797 | 46.907 | 08.03.2011 | C Angst |
| SW15 | CC083 | t_FD_ | g | | 6.862 | 46.944 | 09.09.2007 | C Angst |
| SW16 | CC084 | t_FD_ | g | | 7.050 | 46.860 | 24.09.2007 | C Angst |
| SW17 | CC076 | t_FD_ | g | | 7.922 | 47.364 | 27.06.2004 | C Angst |
| SW18 | CC098 | t_FD_ | f | | 7.114 | 46.985 | 20.11.2009 | C Angst |
| SW19 | CC091 | t_FD_ | f | | 8.169 | 47.469 | 11.05.2008 | C Angst |
| SW20 | CC099 | t_FD_ | f | | 7.101 | 46.980 | 05.12.2009 | C Angst |
| SW21 | CC095 | t_FD_ | f | | 9.165 | 47.531 | 23.06.2009 | C Angst |
| SW22 | CC087 | t_FD_ | f | | 9.067 | 47.440 | 06.04.2008 | C Angst |
| SW23 | CC003 | t_FD_ | f | | 8.280 | 47.731 | 25.12.2011 | B Sättele |
| SW24 | CC075 | t_FD_ | f | | 8.852 | 47.578 | 22.07.2000 | C Angst |
| SW25 | CC092 | t_FD_ | f | | 8.385 | 47.565 | 24.07.2008 | C Angst |
| SW26 | CC004 | t_FD_ | f | | 8.443 | 47.837 | 05.01.2012 | B Sättele |
| SW27 | CC077 | t_FD_ | f | | 8.625 | 47.666 | 21.04.2005 | C Angst |
| SW28 | CC094 | t_FD_ | f | | 7.968 | 47.369 | 12.05.2009 | C Angst |
| SW29 | CC002 | h_FD_ | f | | 8.573 | 47.652 | 16.12.2011 | B Sättele |
| SW30 | CC089 | t_FD_ | f | | 8.298 | 47.591 | 25.04.2008 | C Angst |
| SW31 | CC090 | t_FD_ | f | | 8.101 | 47.564 | 11.05.2008 | C Angst |
| SW32 | CC093 | t_FD_ | f | | 8.237 | 47.611 | 18.08.2008 | C Angst |
| GR01 | CC025 | h_NS_ | r_1_ | | 4.667 | 50.467 | 24.10.2008 | J Michaux |
| GR02 | CC028 | h_NS_ | r_1_ | | 5.370 | 50.340 | 14.07.2009 | J Michaux |
| GR03 | CC026 | h_NS_ | r_1_ | | 4.648 | 50.452 | 26.11.2008 | J Michaux |
| GR04 | CC035 | h_NS_ | g | | 5.836 | 50.165 | 02.03.2010 | J Michaux |
| GR05 | CC041 | h_NS_ | r_1_ | | 4.817 | 50.217 | 2009-2012 | J Michaux |
| GR06 | CC033 | h_NS_ | g | | 5.933 | 50.183 | 02.03.2010 | J Michaux |
| GR07 | CC045 | h_NS_ | g | | 5.845 | 49.862 | 19.03.2010 | J Michaux |
| GR08 | CC027 | h_NS_ | r_1_ | | 5.575 | 49.976 | 01.12.2008 | J Michaux |
| GR09 | CC051 | h_NS_ | r_1_ | | 6.327 | 50.152 | 2009-2012 | J Michaux |
| GR10 | CC024 | h_NS_ | r_1_ | | 5.517 | 50.017 | 22.10.2008 | J Michaux |
| GR11 | CC040 | h_NS_ | r_1_ | | 4.480 | 50.528 | 05.04.2010 | J Michaux |
| GR12 | CC042 | h_NS_ | f | | 6.024 | 50.405 | 18.03.2010 | J Michaux |
| GR13 | CC039 | h_NS_ | r_1_ | | 6.127 | 50.279 | 11.03.2010 | J Michaux |
| GR14 | CC043 | h_NS_ | r_1_ | | 5.915 | 50.284 | 12.03.2010 | J Michaux |
| GR15 | CC053 | h_NS_ | r_1_ | | 6.326 | 50.153 | 2009-2012 | J Michaux |
| GR16 | CC036 | h_NS_ | r_1_ | | 6.017 | 50.422 | 02.03.2010 | J Michaux |
| GR17 | CC029 | h_NS_ | r_1_ | | 5.647 | 50.708 | 19.03.2010 | J Michaux |
| GR18 | CC031 | h_NS_ | r_1_ | | 6.026 | 50.427 | 18.03.2010 | J Michaux |
| GR19 | CC052 | h_NS_ | g | | 6.329 | 50.152 | 2009-2012 | J Michaux |
| GR20 | CC030 | h_NS_ | f | | 5.580 | 50.632 | 19.03.2010 | J Michaux |
| GR21 | CC015 | h_NS_ | a_1_ | | 6.884 | 49.621 | 01.03.2012 | S Venske |
| GR22 | CC103 | h_NS_ | r_1_ | | 6.480 | 50.663 | 12.06.2012 | L Dalbeck |
| GR23 | CC102 | t_FD_ | e | | 6.278 | 50.972 | 05.02.2012 | L Dalbeck |
| GR24 | CC101 | t_FD_ | r_1_ | | 6.485 | 50.653 | 04.01.2008 | L Dalbeck |
| GR25 | CC044 | h_NS_ | c | | 5.984 | 50.133 | 23.10.2009 | J Michaux |
| GR26 | CB007 | t_S_ | c | | 6.172 | 50.138 | 24.02.2011 | S Venske |
| GR27 | CB008 | t_S_ | c | | 6.176 | 50.137 | 02.03.2011 | S Venske |
| GR28 | CB021 | t_S_ | c | | 6.212 | 50.080 | 06.04.2011 | S Venske |
| GR29 | CB022 | t_S_ | c | | 6.246 | 50.113 | 07.04.2011 | S Venske |
| GR30 | CB023 | t_S_ | c | | 6.220 | 50.077 | 23.03.2011 | S Venske |
| GR31 | CC012 | t_S_ | c | | 6.316 | 50.176 | 18.11.2011 | S Venske |
| GR32 | CC013 | t_S_ | c | | 6.284 | 50.206 | 20.11.2011 | S Venske |
| GR33 | CC020 | t_S_ | c | | 6.465 | 50.249 | 11.03.2012 | S Venske |
| GR34 | CC021 | t_S_ | c | | 6.414 | 50.236 | 03.03.2012 | S Venske |
| GR35 | CC037 | h_NS_ | c | | 6.107 | 50.252 | 25.02.2010 | J Michaux |
| GR36 | CC048 | h_NS_ | c | | 6.303 | 50.155 | 09.12.2009 | J Michaux |
| GR37 | CC050 | h_NS_ | c | | 6.283 | 50.196 | 2009-2012 | J Michaux |
| GR38 | CC054 | h_NS_ | c | | 6.273 | 50.258 | 2009-2012 | J Michaux |
| GR39 | CC059 | h_NS_ | c | | 6.331 | 50.150 | 03.04.2009 | J Michaux |
| GR40 | CC061 | h_NS_ | c | | 6.331 | 50.150 | 03.04.2009 | J Michaux |
| GR41 | CC106 | t_S_ | c | | 6.273 | 50.263 | 01.05.2012 | S Venske |
| GR42 | CC107 | t_S_ | c | | 6.332 | 50.297 | 01.05.2012 | S Venske |
| GR43 | CC108 | t_S_ | c | | 6.429 | 50.202 | 01.04.2012 | S Venske |
| GR44 | CC060 | h_NS_ | c | | 6.331 | 50.152 | 03.04.2009 | J Michaux |

NS (Noninvasive Sample):

Hair samples collected with barbed wire hair traps without animal handling. The setting up of hair traps was permitted by “Regierungspräsidium Darmstadt, Hessen”, Germany (Region HE), Walloon Environment Ministry, Belgium, and the Ministry for Nature Conservation from Luxembourg (Region GR).

FD (Found Dead):

Beavers found dead in the wild (traffic mortality, illness, died of old age). Tissue or hair samples of the dead found beavers were collected by local beaver experts, veterinarians and police.

C (Culling)

Culling of beavers is the exception in Germany. No beavers were killed for our study. Thirteen beavers were killed in region BB with permission of the appropriate nature conservation authority, Bavaria. Beavers found in sewage plants or canals of power plants were killed according to the legal ordinance of the “AAV-Artenschutzrechtliche Ausnahmeverordnung”, Bavaria, Germany.

S (Steralisation):

This concerns samples which originated from sterilisation of the invasive *C. canadensis*. After sterilisation, beavers were released to their former territory or transferred to zoos. Sterilisation was executed by the “Gesellschaft für Naturschutz und Ornithologie Rheinland Pfalz e.V.”, Germany, with permission from the “Ministerium für Umwelt, Landwirtschaft, Ernährung, Weinbau und Forsten in Rheinland-Pfalz”, Germany. Live-trapping for species identification was permitted by “Struktur- und Genehmigungsdirektion Nord, Koblenz”, Germany.
