# Supplementary material for: The Genetic Legacy of Multiple Beaver Reintroductions in Central Europe
Source: PLoS One. 2014 May 14;9(5):e97619. doi: 10.1371/journal.pone.0097619 (PMC4020922; doi:10.1371/journal.pone.0097619)
Supplement: Table S2 — Overview of successfully analysed samples for the five regions. (DOCX) [file pone.0097619.s002.docx]

**Supplementary Table S2** Overview of successfully analysed samples for the five regions. *=data set contains *C. canadensis* (Belgium n=1, Rhineland-Palatinate n=18, Luxemburg n=1).

| Region | Sample origin | Number of samples | | |
| --- | --- | --- | --- | --- |
|  |  | tissue | hair | total |
| HE | Hesse, border to Bavaria | 20 | 22 | 42 |
| EG | Brandenburg, border to Thuringia, border to Saxony | 50 | 3 | 53 |
| BB | Bavaria, Baden-Württemberg | 63 | 1 | 64 |
| SW | Switzerland, border to Baden-Württemberg, border to France | 31 | 1 | 32 |
| GR | Belgium*, Rhineland-Palatinate*, Luxemburg*, Northrhine-Westphalia | 14 | 30 | 44 |
| Total |  | 178 | 57 | 235 |
