# Supplementary material for: The Genetic Legacy of Multiple Beaver Reintroductions in Central Europe
Source: PLoS One. 2014 May 14;9(5):e97619. doi: 10.1371/journal.pone.0097619 (PMC4020922; doi:10.1371/journal.pone.0097619)
Supplement: Table S3 — Error rates of the 57 analysed hair samples. (DOCX) [file pone.0097619.s003.docx]

Supplementary Table S3 Error rates of the 57 analysed hair samples.*=Marker not usable for *C. canadensis* (which explains the low success rate for CF5 and CF41). Genotyping error is divided into allelic dropout (where an allele that is present in reality is not scored during genotyping) and false allele (where an allele that is in reality not present appears in the genotype).

| Locus | PCR success [%] | Dropout [%] | False [%] |
| --- | --- | --- | --- |
| CF32 | 99 | - | - |
| Cca18 | 98 | 4 | - |
| Cca13 | 98 | - | 2 |
| CF33 | 99 | 2 | 2 |
| CF44 | 97 | 1 | - |
| CF7 | 95 | - | - |
| Cca4 | 91 | 6 | 6 |
| Cca8 | 84 | 4 | 3 |
| CF6 | 100 | 3 | - |
| CF31 | 98 | 1 | 6 |
| CF19 | 99 | 2 | - |
| CF5* | 84 | 12 | - |
| CF41* | 86 | - | 1 |
| Mean | 94.5 | 2.7 | 1.5 |
