# Supplementary material for: The Genetic Legacy of Multiple Beaver Reintroductions in Central Europe
Source: PLoS One. 2014 May 14;9(5):e97619. doi: 10.1371/journal.pone.0097619 (PMC4020922; doi:10.1371/journal.pone.0097619)
Supplement: Table S4 — Microsatellites evaluated and used in this study. (DOCX) [file pone.0097619.s004.docx]

**Supplementary Table S4** Microsatellites evaluated and used in this study.

| Locus name | Length (bp) | Repeat | Label | Multiplex | Forward/ reverse primer | Accession-number | Problems in analysis | Considered in final dataset |
| --- | --- | --- | --- | --- | --- | --- | --- | --- |
| Cca13^1^ | 264-278 | GT(11)GT(7) | VIC | A | CCCTAGACTTTGATTATACGG AGGTTGCCTAGAGAGAGGTGTG | EF524506 | no | yes |
| Cca18^1^ | 209-219 | CT(10) | 6FAM | A | CTGCTGTGGGATCTTGGATT TGGTATGTGCTACACAGAAAACAA | EF5245010 | no | yes |
| CF31^2^ | 210-224 | (GT)22 | NED | A | TCCCTCAGGCTTTAATTGGA TCTCGAGCCCCTATCTTGAA | HQ698347 | no | yes |
| CF32^2^ | 135-163 | (TG)16 | 6FAM | A | CAGTTTTGTTCTCTCTCTCACTATGAA GGCTCAAAAAGTCAAAGGTCA | HQ698348 | no | yes |
| CF33^2^ | 181-219 | (TG)20 | NED | A | TGCCACCCTAACAAATAGGTG GTTTTGCTCATCAGCCCTGT | HQ698349 | no | yes |
| CF05^2^ | 214-217 | (TG)15 | 6FAM | B | GGAAATATTTAAAGGGTCGAATG TTGCAGTTTCTTGGAACACG | HQ698339 | no | yes |
| CF07^2^ | 96-108 | (AC)10 | PET | C | CTTTTGCCACACTCAACACAA TTCCTAGGCAGAAATCAGGAA | HQ698341 | no | yes |
| CF44^2^ | 203-219 | (TG)20 | PET | C | GGGGAAAGGGAGAGGAGTTT TAATCCTATCCCCCAAGTCG | HQ698351 | no | yes |
| Cca4^1^ | 355-396 | AC(17) | 6FAM | D | GATTTCAGACACAGCCACCA AGTGATGGGATTGAACTCCAG | EF524501 | no | yes |
| Cca8^1^ | 378-410 | GATA(12) | VIC | D | GGGCTCAGAGGAAAAAGGAG GATCAGGCAAAAGGCTGGTA | EF524503 | no | yes |
| CF06^2^ | 130-142 | (GT)14 | NED | D | TGTGGCCTGTAATACGAAAAG TCAGATGTTCAAGACCACCAA | HQ698340 | no | yes |
| CF19^2^ | 177-191 | (CA)16 | PET | D | AGTGGGCTGCCATTGTTAAG TCTGCACAGTGTTCATGCTG | HQ698344 | no | yes |
| CF41^2^ | 100-108 | (CA)17 | PET | D | CAACCACTCCCACCCACTC TGTCTGCCTGGTAAGCATGA | HQ698350 | no | yes |
| CF17^2^ | 197-226 | (GT)13 | NED | B | CCAAGAGGGCTGTCTCATGT CGTTGTGCTTGCTAGGTGAG | HQ698342 | results inconsistent | no |
| CF21^2^ | 199-201 | (TG)9 | PET | B | CATGGGGTGGGGGTATTATC ATCCTTTCTTGGTCCTGCAA | HQ698345 | results inconsistent | no |
| CF49^2^ | 132-136 | (CA)7 | 6FAM | B | GTGCCCAGCATCGAGAAGTA CCCCTCTGCTGTGTGCTAGT | HQ698353 | no reliable amplification | no |
| Cca5^1^ | 144-182 | CT(21) | VIC | C | TGCTTTCATCTGCTCTATGAAAAT CAGTGATGAAGGGAAGAGGAA | EF524502 | results inconsistent | no |
| CF18^2^ | 209-240 | (AC)15 | NED | C | TGTCTCCCAAATGGACTTCA CCGTCTTCAAGCCATAAACC | HQ698343 | results inconsistent | no |
| CF30^2^ | 202-204 | (AC)17 | VIC | D | GCCCATGCTCTCAGCATTAT TGCGCACATGTATATGACTCAC | HQ698346 | results inconsistent | no |
| CF48^2^ | 191–199 | (GT)13 | 6FAM | no | AGTGGTTGCCCAAAATGAAC GGAGTGGCTTAAGGTGTTCG | HQ698352 | no reliable amplification | no |
| Cca13^1^ | 264–272 | GT(11)GT(7) | VIC | no | CCCTAGACTTTGATTATACGG AGGTTGCCTAGAGAGAGGTGTG | EF524506 | no reliable amplification | no |
| Cca112^3^ | 192–194 | (GT)18 | VIC | no | CCCAAAAAAGATGTTCTT GACTTTGCTGGTTTAGAGGTTG | EU704006 | no reliable amplification | no |
| Cca56^3^ | 242–250 | (CA)9TATA(CA)19 | NED | no | GCAGAGCACCAATAAAATCC CTGGACTCTTGGAAACGCC | EU704002 | no reliable amplification | no |
| Cca76^3^ | 157–189 | (GT)20 | PET | no | CCCATAGAACCCAAAGCAG CAACTGACTGCAAATAGCTACAGC | EU704004 | no reliable amplification | no |
| Cca92^3^ | 196–214 | (CA)20 | NED | no | TCCTTGACTCTGGGCATG CAGAGCTTCCACAGTATCTGG | EU704005 | no reliable amplification | no |

^1^ Crawford JC, Liu Z, Nelson TA, Nielsen CK, Bloomquist CK (2008) Microsatellite analysis of mating and kinship in beavers (*Castor canadensis*). J Mamm 89: 575-581.

^2^ Frosch C, Haase P, Nowak C (2011) First set of microsatellite markers for genetic characterization of the Eurasian beaver (*Castor fiber*) based on tissue and hair samples. Eur J Wildlife Res 57: 679-682.

^3^ Pelz-Serrano K, Munguia-Vega A, Piaggio AJ, Neubaum M, Munclinger P, et al. (2009) Development of nine new microsatellite loci for the American beaver, *Castor canadensis* (Rodentia: Castoridae), and cross-species amplification in the European beaver, *Castor fiber*. Mol Ecol Res 9: 551-554.
