# Supplementary material for: The Genetic Legacy of Multiple Beaver Reintroductions in Central Europe
Source: PLoS One. 2014 May 14;9(5):e97619. doi: 10.1371/journal.pone.0097619 (PMC4020922; doi:10.1371/journal.pone.0097619)
Supplement: Table S5 — Observed and expected heterozygosity and deviations from Hardy-Weinberg equilibrium per region and per locus. (DOCX) [file pone.0097619.s005.docx]

**Supplementary Table S5** Observed and expected heterozygosity and deviations from Hardy-Weinberg equilibrium per region/sub-population and per locus.

| Region | Population | Locus | n | H_obs_ | H_exp_ | p |
| --- | --- | --- | --- | --- | --- | --- |
| Region I | combined | **CF32** | **42** | **0.16667** | **0.55049** | ***0.00000** |
| HE |  | Cca18 | 42 | 0.30952 | 0.37952 | 0.03048 |
|  |  | **Cca13** | **42** | **0.26190** | **0.58204** | ***0.00000** |
|  |  | CF33 | 42 | 0.23810 | 0.41480 | 0.00062 |
|  |  | CF44 | 42 | 0.28571 | 0.37751 | 0.02181 |
|  |  | CF7 | 42 | 0.30952 | 0.35427 | 0.40478 |
|  |  | **Cca4** | **41** | **0.34146** | **0.57964** | ***0.00033** |
|  |  | **Cca8** | **42** | **0.35714** | **0.60069** | ***0.00000** |
|  |  | CF6 | 42 | 0.35714 | 0.48279 | 0.00771 |
|  |  | **CF31** | **42** | **0.42857** | **0.61331** | ***0.00003** |
|  |  | CF19 | 42 | 0.09524 | 0.09180 | 1.00000 |
|  |  | **CF5** | **42** | **0.28571** | **0.60040** | ***0.00000** |
|  |  | CF41 | 40 | 0.47500 | 0.52943 | 0.41369 |
|  | Subpopulation I | CF32 | no var. |  |  |  |
|  |  | Cca18 | 21 | 0.09524 | 0.09292 | 1.00000 |
|  |  | Cca13 | no var. |  |  |  |
|  |  | CF33 | 21 | 0.04762 | 0.04762 | 1.00000 |
|  |  | CF44 | 21 | 0.09524 | 0.09292 | 1.00000 |
|  |  | CF7 | 21 | 0.19048 | 0.17654 | 1.00000 |
|  |  | Cca4 | 20 | 0.10000 | 0.09744 | 1.00000 |
|  |  | Cca8 | 21 | 0.09524 | 0.09292 | 1.00000 |
|  |  | CF6 | 21 | 0.14286 | 0.13589 | 1.00000 |
|  |  | CF31 | 21 | 0.14286 | 0.13821 | 1.00000 |
|  |  | CF19 | no var. |  |  |  |
|  |  | CF5 | 21 | 0.19048 | 0.53310 | 0.00122 |
|  |  | CF41 | 21 | 0.57143 | 0.51103 | 0.67521 |
|  | Subpopulation II | CF32 | 14 | 0.28571 | 0.50529 | 0.08993 |
|  |  | Cca18 | 14 | 0.57143 | 0.59259 | 0.00216 |
|  |  | Cca13 | 14 | 0.42857 | 0.55291 | 0.42261 |
|  |  | CF33 | 14 | 0.64286 | 0.67989 | 0.04399 |
|  |  | CF44 | 14 | 0.42857 | 0.58201 | 0.16434 |
|  |  | CF7 | 14 | 0.42857 | 0.50794 | 0.62111 |
|  |  | Cca4 | 14 | 0.50000 | 0.58730 | 0.22987 |
|  |  | Cca8 | 14 | 0.57143 | 0.60582 | 0.40122 |
|  |  | CF6 | 14 | 0.64286 | 0.65873 | 0.58756 |
|  |  | CF31 | 14 | 0.71429 | 0.54762 | 0.26878 |
|  |  | CF19 | 14 | 0.28571 | 0.25397 | 1.00000 |
|  |  | CF5 | 14 | 0.42857 | 0.50794 | 0.62073 |
|  |  | CF41 | 12 | 0.50000 | 0.42029 | 1.00000 |
| Region II | combined | CF32 | 53 | 0.35849 | 0.44385 | 0.00206 |
| EG |  | **Cca18** | **53** | **0.33962** | **0.46361** | ***0.00000** |
|  |  | **Cca13** | **53** | **0.22642** | **0.41815** | ***0.00001** |
|  |  | CF33 | 53 | 0.18868 | 0.29739 | 0.01144 |
|  |  | **CF44** | **52** | **0.21154** | **0.39843** | ***0.00000** |
|  |  | CF7 | no var. |  |  |  |
|  |  | Cca4 | 52 | 0.23077 | 0.24552 | 0.27367 |
|  |  | Cca8 | 52 | 0.25000 | 0.42494 | 0.00446 |
|  |  | CF6 | 53 | 0.16981 | 0.15687 | 1.00000 |
|  |  | CF31 | 53 | 0.33962 | 0.47565 | 0.00525 |
|  |  | CF19 | 53 | 0.24528 | 0.37305 | 0.00471 |
|  |  | CF5 | 53 | 0.20755 | 0.58814 | 0.00000 |
|  |  | CF41 | 51 | 0.56863 | 0.53970 | 0.06799 |
|  | Subpopulation I | CF32 | 30 | 0.03333 | 0.03333 | 1.00000 |
|  |  | **Cca18** | **30** | **0.10000** | **0.18814** | ***0.00005** |
|  |  | Cca13 | 30 | 0.00000 | 0.06554 | 0.01688 |
|  |  | CF33 | 30 | 0.16667 | 0.15876 | 1.00000 |
|  |  | CF44 | no var. |  |  |  |
|  |  | CF7 | no var. |  |  |  |
|  |  | Cca4 | no var. |  |  |  |
|  |  | Cca8 | no var. |  |  |  |
|  |  | CF6 | no var. |  |  |  |
|  |  | CF31 | 30 | 0.06667 | 0.06610 | 1.00000 |
|  |  | CF19 | no var. |  |  |  |
|  |  | **CF5** | **30** | **0.16667** | **0.52034** | ***0.00000** |
|  |  | CF41 | 28 | 0.75000 | 0.50844 | 0.02046 |
|  | Subpopulation II | CF32 | 16 | 0.87500 | 0.71169 | 0.52734 |
|  |  | Cca18 | 16 | 0.62500 | 0.44355 | 0.23301 |
|  |  | Cca13 | 16 | 0.50000 | 0.59476 | 0.62182 |
|  |  | CF33 | 16 | 0.06250 | 0.41734 | 0.00152 |
|  |  | CF44 | 15 | 0.66667 | 0.69195 | 0.05416 |
|  |  | CF7 | no var. |  |  |  |
|  |  | Cca4 | 16 | 0.62500 | 0.56653 | 0.90148 |
|  |  | Cca8 | 16 | 0.50000 | 0.40121 | 0.63403 |
|  |  | CF6 | 16 | 0.56250 | 0.41734 | 0.25695 |
|  |  | CF31 | 16 | 0.75000 | 0.63508 | 0.83609 |
|  |  | CF19 | 16 | 0.62500 | 0.60282 | 1.00000 |
|  |  | CF5 | 16 | 0.31250 | 0.35282 | 1.00000 |
|  |  | CF41 | 16 | 0.31250 | 0.49194 | 0.01093 |
| Region III | combined | CF32 | 62 | 0.54839 | 0.54275 | 0.63525 |
| BB |  | Cca18 | 64 | 0.26562 | 0.25898 | 1.00000 |
|  |  | Cca13 | 64 | 0.51562 | 0.48905 | 0.79711 |
|  |  | **CF33** | **64** | **0.26562** | **0.58551** | ***0.00000** |
|  |  | CF44 | 64 | 0.59375 | 0.59313 | 1.00000 |
|  |  | **CF7** | **64** | **0.48438** | **0.50627** | ***0.00037** |
|  |  | Cca4 | 61 | 0.42623 | 0.54545 | 0.03585 |
|  |  | Cca8 | 60 | 0.66667 | 0.65196 | 0.61750 |
|  |  | CF6 | 64 | 0.54688 | 0.66129 | 0.03961 |
|  |  | CF31 | 64 | 0.46875 | 0.49680 | 0.21281 |
|  |  | CF19 | 64 | 0.43750 | 0.39321 | 0.52100 |
|  |  | CF5 | 64 | 0.34375 | 0.47982 | 0.03466 |
|  |  | CF41 | 63 | 0.34921 | 0.40178 | 0.03775 |
|  | Subpopulation I | CF32 | 12 | 0.83333 | 0.68116 | 0.32580 |
|  |  | Cca18 | 13 | 0.15385 | 0.14769 | 1.00000 |
|  |  | Cca13 | 13 | 0.38462 | 0.32308 | 1.00000 |
|  |  | CF33 | no var. |  |  |  |
|  |  | CF44 | 13 | 0.61538 | 0.62462 | 0.88516 |
|  |  | CF7 | 13 | 0.53846 | 0.40923 | 0.49920 |
|  |  | Cca4 | 13 | 0.30769 | 0.59077 | 0.02452 |
|  |  | Cca8 | 13 | 0.53846 | 0.49538 | 1.00000 |
|  |  | CF6 | 13 | 0.46154 | 0.49231 | 0.50642 |
|  |  | CF31 | 13 | 0.92308 | 0.51692 | 0.00566 |
|  |  | CF19 | 13 | 0.15385 | 0.14769 | 1.00000 |
|  |  | CF5 | 13 | 0.38462 | 0.32308 | 1.00000 |
|  |  | CF41 | 13 | 0.15385 | 0.14769 | 1.00000 |
|  | Subpopulation II | CF32 | 15 | 0.53333 | 0.48046 | 1.00000 |
|  |  | Cca18 | 15 | 0.13333 | 0.12874 | 1.00000 |
|  |  | Cca13 | 15 | 0.46667 | 0.43448 | 1.00000 |
|  |  | CF33 | 15 | 0.40000 | 0.61379 | 0.06250 |
|  |  | CF44 | 15 | 0.60000 | 0.50805 | 0.80716 |
|  |  | CF7 | 15 | 0.60000 | 0.55862 | 0.01970 |
|  |  | Cca4 | 13 | 0.23077 | 0.21231 | 1.00000 |
|  |  | Cca8 | 12 | 0.75000 | 0.65217 | 0.40565 |
|  |  | CF6 | 15 | 0.46667 | 0.60690 | 0.47949 |
|  |  | CF31 | 15 | 0.26667 | 0.40230 | 0.06852 |
|  |  | CF19 | 15 | 0.46667 | 0.43448 | 1.00000 |
|  |  | CF5 | 15 | 0.06667 | 0.06667 | 1.00000 |
|  |  | CF41 | 15 | 0.80000 | 0.66207 | 0.59749 |
| Region IV | combined | CF32 | 28 | 0.39286 | 0.72273 | 0.00096 |
| SW |  | Cca18 | 32 | 0.18750 | 0.22222 | 0.39248 |
|  |  | Cca13 | 32 | 0.31250 | 0.30952 | 1.00000 |
|  |  | **CF33** | **32** | **0.31250** | **0.56200** | ***0.00015** |
|  |  | **CF44** | **32** | **0.31250** | **0.70040** | ***0.00001** |
|  |  | **CF7** | **32** | **0.18750** | **0.50595** | ***0.00032** |
|  |  | Cca4 | 31 | 0.51613 | 0.64781 | 0.00727 |
|  |  | **Cca8** | **31** | **0.32258** | **0.73876** | ***0.00000** |
|  |  | **CF6** | **32** | **0.25000** | **0.69395** | ***0.00000** |
|  |  | **CF31** | **32** | **0.34375** | **0.60466** | ***0.00000** |
|  |  | CF19 | 32 | 0.31250 | 0.30952 | 1.00000 |
|  |  | CF5 | 31 | 0.22581 | 0.45003 | 0.00140 |
|  |  | CF41 | 30 | 0.30000 | 0.50113 | 0.02290 |
|  | Subpopulation I | CF32 | 10 | 0.50000 | 0.72105 | 0.30607 |
|  |  | Cca18 | 12 | 0.41667 | 0.43116 | 1.00000 |
|  |  | Cca13 | 12 | 0.50000 | 0.46377 | 1.00000 |
|  |  | CF33 | 12 | 0.58333 | 0.53623 | 0.82527 |
|  |  | CF44 | 12 | 0.33333 | 0.42391 | 0.27763 |
|  |  | CF7 | no var. |  |  |  |
|  |  | Cca4 | 12 | 0.66667 | 0.52174 | 0.56338 |
|  |  | Cca8 | 12 | 0.33333 | 0.46377 | 0.51762 |
|  |  | CF6 | 12 | 0.16667 | 0.50725 | 0.02873 |
|  |  | CF31 | 12 | 0.66667 | 0.52174 | 0.56242 |
|  |  | CF19 | 12 | 0.33333 | 0.28986 | 1.00000 |
|  |  | CF5 | 12 | 0.41667 | 0.51812 | 0.69784 |
|  |  | CF41 | 12 | 0.58333 | 0.56159 | 0.78462 |
|  | Subpopulation II | CF32 | 13 | 0.30769 | 0.39692 | 0.52270 |
|  |  | Cca18 | no var. |  |  |  |
|  |  | Cca13 | 15 | 0.06667 | 0.06667 | 1.00000 |
|  |  | CF33 | 15 | 0.06667 | 0.06667 | 1.00000 |
|  |  | CF44 | 15 | 0.26667 | 0.45977 | 0.23179 |
|  |  | CF7 | 15 | 0.20000 | 0.28736 | 0.32700 |
|  |  | Cca4 | 14 | 0.28571 | 0.26190 | 1.00000 |
|  |  | **Cca8** | **14** | **0.14286** | **0.51852** | ***0.00037** |
|  |  | CF6 | 15 | 0.13333 | 0.34253 | 0.01933 |
|  |  | CF31 | no var. |  |  |  |
|  |  | CF19 | 15 | 0.33333 | 0.37011 | 1.00000 |
|  |  | CF5 | no var. |  |  |  |
|  |  | CF41 | no var. |  |  |  |
| Region V |  | CF32 | 28 | 0.39286 | 0.72273 | 0.00068 |
| GR |  | Cca18 | 32 | 0.18750 | 0.22222 | 0.38926 |
|  |  | Cca13 | 32 | 0.31250 | 0.30952 | 1.00000 |
|  |  | **CF33** | **32** | **0.31250** | **0.56200** | ***0.00021** |
|  |  | **CF44** | **32** | **0.31250** | **0.70040** | ***0.00002** |
|  |  | CF7 | 32 | 0.18750 | 0.50595 | 0.00039 |
|  |  | Cca4 | 31 | 0.51613 | 0.64781 | 0.00675 |
|  |  | **Cca8** | **31** | **0.32258** | **0.73876** | ***0.00000** |
|  |  | **CF6** | **32** | **0.25000** | **0.69395** | ***0.00000** |
|  |  | **CF31** | **32** | **0.34375** | **0.60466** | ***0.00000** |
|  |  | CF19 | 32 | 0.31250 | 0.30952 | 1.00000 |
|  |  | CF5 | 31 | 0.22581 | 0.45003 | 0.00117 |
|  |  | CF41 | 30 | 0.30000 | 0.50113 | 0.02283 |
| *C. canadensis* |  | CF32 | 20 | 0.85000 | 0.73462 | 0.19562 |
|  |  | **Cca18** | **20** | **0.00000** | **0.46667** | ***0.00000** |
|  |  | Cca13 | 20 | 0.05000 | 0.05000 | 1.00000 |
|  |  | **CF33** | **19** | **0.36842** | **0.64154** | ***0.00000** |
|  |  | CF44 | no var. |  |  |  |
|  |  | CF7 | no var. |  |  |  |
|  |  | Cca4 | 20 | 0.55000 | 0.49872 | 1.00000 |
|  |  | Cca8 | 18 | 0.61111 | 0.50000 | 0.62423 |
|  |  | CF6 | 20 | 0.55000 | 0.50128 | 1.00000 |
|  |  | CF31 | 19 | 0.78947 | 0.68279 | 0.79582 |
|  |  | CF19 | 20 | 0.60000 | 0.57949 | 0.13879 |
|  |  | CF5 | N/A |  |  |  |
|  |  | CF41 | N/A |  |  |  |

n = sample size

no var. = locus monomorphic in the respective region/sub-population

* = locus statistically not in HWE, additionally highlighted in boldface.
