# Supplementary material for: The Genetic Legacy of Multiple Beaver Reintroductions in Central Europe
Source: PLoS One. 2014 May 14;9(5):e97619. doi: 10.1371/journal.pone.0097619 (PMC4020922; doi:10.1371/journal.pone.0097619)
Supplement: Table S6 — Overview of haplotypes. (DOCX) [file pone.0097619.s006.docx]

**Supplementary Table S6** Overview haplotypes. Number of beaver samples with the haplotypes c, a_1_, g, r_1_, f and e for every region and in total.

| haplotype | *c* | *a_1_* | *g* | *r_1_* | *f* | *e* |
| --- | --- | --- | --- | --- | --- | --- |
| (sub)species | *C. canadensis* | *C. f. albicus* | *C. f. galliae* | *C. f. sp.* | *C. f. fiber* | *C. f. sp.* |
| HE | 0 | 28 | 1 | 13 | 0 | 0 |
| EG | 0 | 44 | 0 | 9 | 0 | 0 |
| BB | 0 | 0 | 24 | 39 | 1 | 0 |
| SW | 0 | 0 | 17 | 0 | 15 | 0 |
| GR | 20 | 1 | 4 | 16 | 2 | 1 |
| total | 20 | 73 | 46 | 77 | 18 | 1 |
