# Supplementary material for: The Genetic Legacy of Multiple Beaver Reintroductions in Central Europe
Source: PLoS One. 2014 May 14;9(5):e97619. doi: 10.1371/journal.pone.0097619 (PMC4020922; doi:10.1371/journal.pone.0097619)
Supplement: Table S7 — Allelic richness across regions. (DOCX) [file pone.0097619.s007.docx]

Supplementary Table S7 Allelic richness across regions. Uncorrected (A) and corrected (A_corr_) allelic richness are displayed for all combined *C. fiber* individuals in this study, as well for the specific regions. Corrections for sample size were conducted by rarefaction in HP-rare 1.1 [1], to a minimum sample size of 27 individuals. Among regions the means of allelic richness across loci are generally similar.

|  | *C. fiber* | | HE | | EG | | BB | | SW | | GR | |
| --- | --- | --- | --- | --- | --- | --- | --- | --- | --- | --- | --- | --- |
| Locus | A | **A_corr_** | A | **A_corr_** | A | **A_corr_** | A | **A_corr_** | A | **A_corr_** | A | **A_corr_** |
| CF32 | 7 | **5.58** | 4 | **3.99** | 5 | **4.78** | 4 | **3.45** | 5 | **5.00** | 5 | **5.00** |
| Cca18 | 4 | **3.41** | 3 | **3.00** | 4 | **3.80** | 3 | **2.69** | 2 | **2.00** | 2 | **2.00** |
| Cca13 | 5 | **4.20** | 4 | **3.67** | 5 | **4.21** | 2 | **2.00** | 2 | **2.00** | 2 | **2.00** |
| CF33 | 6 | **5.39** | 5 | **4.33** | 4 | **3.68** | 6 | **5.30** | 4 | **3.99** | 4 | **3.99** |
| CF44 | 6 | **5.02** | 3 | **2.89** | 5 | **4.07** | 4 | **3.94** | 4 | **4.00** | 4 | **4.00** |
| CF7 | 3 | **2.43** | 2 | **2.00** | 1 | **1.00** | 3 | **2.90** | 2 | **2.00** | 2 | **2.00** |
| Cca4 | 5 | **4.44** | 5 | **4.59** | 4 | **3.53** | 5 | **4.29** | 3 | **3.00** | 3 | **3.00** |
| Cca8 | 7 | **6.15** | 4 | **3.97** | 3 | **2.79** | 5 | **4.83** | 4 | **4.00** | 4 | **4.00** |
| CF6 | 4 | **3.57** | 4 | **3.67** | 2 | **2.00** | 3 | **3.00** | 4 | **4.00** | 4 | **4.00** |
| CF31 | 7 | **4.97** | 4 | **3.67** | 5 | **4.48** | 4 | **3.42** | 3 | **3.00** | 3 | **3.00** |
| CF19 | 3 | **2.72** | 2 | **1.99** | 3 | **2.99** | 2 | **2.00** | 2 | **2.00** | 2 | **2.00** |
| CF5 | 3 | **3.00** | 3 | **3.00** | 3 | **3.00** | 2 | **2.00** | 3 | **2.99** | 3 | **2.99** |
| CF41 | 3 | **2.99** | 3 | **2.91** | 3 | **2.99** | 3 | **2.97** | 3 | **3.00** | 3 | **3.00** |
| mean | 4.85 | **4.14** | 3.54 | **3.36** | 3.62 | **3.33** | 3.54 | **3.29** | 3.15 | **3.15** | 3.15 | **3.15** |
| s.d. | 1.63 | **1.16** | 0.97 | **0.78** | 1.03 | **1.00** | 1.27 | **1.02** | 0.99 | **0.95** | 0.99 | **0.95** |

A = allelic richness: number of alleles observed at a locus

A_corr_ = alleleic richness normalised to a sample size of 27 (the lowest in the five groups) by rarefaction (HP-rare 1.1) [1].

s.d. = standard deviation

**Supporting reference**

1. Kalinowski ST (2005) HP-RARE 1.0: a computer program for performing rarefaction on measures of allelic richness. Mol Ecol Notes 5: 187-189.
