# Supplementary material for: The Genetic Legacy of Multiple Beaver Reintroductions in Central Europe
Source: PLoS One. 2014 May 14;9(5):e97619. doi: 10.1371/journal.pone.0097619 (PMC4020922; doi:10.1371/journal.pone.0097619)
Supplement: Text S1 — Additional text describing details regarding the beaver population history in each region. (DOCX) [file pone.0097619.s009.docx]

**Supplementary Text S1** Detailed description of study areas.

Region HE (I)

In 1970 the preparation for the resettlement of the Eurasian beaver in Hesse began. The aim of the project was the reintroduction of the endemic *C. fiber albicus*. During the Cold War period from 1949-1990 the German relict population was situated in the German Democratic Republic, which complicated transport of autochthonous *C. f. albicus* beaver individuals to Western Germany [1]. In 1987 and 1988, three beaver pairs and 12 individuals were reintroduced, respectively, to the ‘Sinn’ and ‘Jossa’ river systems (in the conservation area ‘Westerngrund’ in the ‘Spessart’ mountain range) from the Elbe relict population in Eastern Germany. The reintroduction was successful and the population grew slowly but steadily to ~175 individuals. Aside from the Spessart population, the first individuals were recorded in 2000/2001 at ‘Fliede’ river in the administrative district ‘Fulda’. In 2007 the first beaver observation was made in the river systems of ‘Nidder’ and ‘Nidda’ in the ‘Wetterau’ region [2]. In total, 87 occupied beaver territories were recorded in 2012, equating to an overall population size of 287 individuals in Hesse [2]. Since 1990, beavers from the reintroduced Hessian population resettled to the Bavarian administrative region of ‘Unterfranken’. In 2012, around 687 individuals were estimated in this region [3].

Region EG (II)

By the end of the 19th century only a small relict population of around 200 individuals survived at the Elbe river system in Saxony, Saxony-Anhalt and Brandenburg in the lower Elbe-Havel area [1,4]. Due to strong protection at the beginning of the 20th century the Elbe beaver population started to spread, but the dispersal was restricted due to limited habitats and infectious diseases [4]. The relict beaver population was supported by several successful reintroductions of the *C. f. albicus* individuals from the Elbe river system: between 1935-1943 in three different locations in the area of ‘Schorfheide’ (‘Pinnowseen’, ‘Großer Lubowsee’, ‘Werbellinsee’), 1973 in the area of Templin and between 1984-1989 in the region of the ‘Oder’ river [4]. The current population size is ~2,800 individuals. In the main dispersal areas, including the watersheds of ‘Schwarze Elster’, ‘Havel’ and ‘Rhin’ all available potential habitats are widely occupied. In these areas the population size is stable or slightly declining. The watersheds of ‘Spree’, ‘Dahme’, ‘Uecker’ and the river systems of ‘Plane’, ‘Nuthe’, ‘Stepenitz’ and ‘Loecknitz’ are only sparsely populated with further potential for resettlement.

Additional beavers from Poland dispersed to Brandenburg. After the Second World War beavers appeared in Eastern Poland and beavers from bordering countries such as Russia, Lithuania and Belarus migrated to Poland [5]. In 1958 beavers were bred in the Research Station of the Polish Academy of Sciences at Popielno and since 1974 they were reintroduced to different parts of Poland [6,7]. The foundation of this farm consisted of beavers from the Russian beaver station in Voronezh next to the eponymous river [8]. Due to many successful reintroductions beavers can now be found across Poland with an estimated population size of 40,000 individuals [5].

Region BB (III)

In the 17th century, numerous beavers lived in southern Germany. The following 200 years were marked by an intensive hunting period [9] that resulted in a significantly reduced population. This led to the regional extinction of the beaver at the Upper Rhine and ‘Ill’ near Strasbourg as well as at the ‘Iller’ at the muzzle area at the Danube near Ulm between 1828 and 1834 [10,11]. In 1887 the last beaver was killed at the ‘Amper’ [12]. In 1966 Voronezh beavers were reared and released in ‘Neustadt’ at the Danube [13]. In 1970 several beavers (*C. f. fiber*) from ‘Karlstad’ in Sweden were successfully reintroduced into the region of the river ‘Gründlach’ in the north of ‘Nuremberg’. One year later, two Finnish beavers (*Castor sp.)* and several Swedish beavers (*C. f. fiber*) were released at the ‘Inn’ damming lake, the ‘Lower Inn’ and the ‘Ammersee’ [9]. In the Alsace region, beavers (*C. f. galliae*) have been resettled at the ‘Doller’ near ‘Schönau’ at the same time. The beavers originally derived from the Rhône. Approximately 20 years later in 1993-1995 the reintroduction of Rhône beavers (*C. f. galliae*) has been continued at the ‘Moder’ in the region Lower Rhine [14]. In 1975 two beaver pairs from Karlstad in Sweden (*C. f. fiber*) were released in ‘Altshausen’ in the south-east of Baden-Württemberg but these beavers died within one year [15]. Another resettlement initiative was conducted in 1979 at the dredging lake of ‘Renchen’, where four Rhône beavers (*C. f. galliae*) were released. Two died and the remaining two emigrated to the Alsatian waters near the Upper Rhine [16]. In the mid 1970s, the first migrating beavers in Baden-Württemberg were reported [17]. Here, first settlements of immigrated Rhône beavers (*C. f. galliae*) from Alsace were established in 1989 in ‘Taubergießen’ and 1990 in ‘Laufenburg’ at the upper Rhine through immigrated beavers from Switzerland. At that time, the first Bavarian beavers immigrated to the eastern waters of Baden-Württemberg as well [18]. Currently (2012) about 2,000 beavers are living in Baden-Württemberg and 14,000 in Bavaria.

Region SW (IV)

After the mass eradication at the beginning of the 19th century, recent beaver reintroductions in Switzerland are now considered successful. Between 1956 and 1977, 141 individuals were released at about 30 sites in the Rhine and Rhône river systems [17]. Beavers released on the upper Rhône, around Lake Geneva, originated exclusively from the lower Rhône river system (*C. f. galliae*; France), whereas those released at various sites on the Rhine river system originated from three different populations: lower Rhône, Norway and Russia (‘Voronezh’) [17]. Despite high initial mortality (>50 of the released animals) and the failure of several operations, several beaver populations became established in Switzerland. In 1993, the population was estimated at around 350 beavers: 220 individuals on the Rhône river system and 124 on the Rhine river system (upper Rhine and ‘Aar’ sub-river system, around the ‘Seeland’) [19]. However, populations were fragmented and considered very fragile. No further population increase was expected due to the overall low quality of the riparian environment. Nevertheless, since 1993, following riparian habitat restoration and due to the ecological plasticity of this species, beavers colonised new sites and the population increased both in size and range [20]. The 2008 census count resulted in a population estimate of around 1600 beavers. A remarkable population increase was noticed on the Rhine river system: an estimated 1300 individuals settled along the ‘Aar’ sub-river system, the Rhine and its tributaries and 300 at the Rhône river system, only a few more than 15 years before. The colonisation of small tributaries is still going on and the actual population size for the whole Swiss territory is estimated to some 2000 individuals (winter 2013; C. Angst unpublished data).

Region GR (V)

There were no official beaver reintroductions in the German federal state Rhineland-Palatinate but in the wake of natural resettlement from Saarland, Alsace, Belgium and North Rhine-Westphalia, a small population became established [21]. Additionally, several reintroductions were conducted in the federal state of North Rhine-Westphalia. In 1981, three beaver pairs from the beaver breeding farm in Popielno, Poland, [22] as well as six additional beavers, were reintroduced from Poland to the Eifel region [23]. Between 2002 and 2004, 26 *C. f. albicus* from the Elbe river system were reintroduced to the lower Rhine in the region of Wesel [24].

Between 1998 and 2002 at least four beavers from the Elbe River system and 97 beavers from Bavaria were released [25] in Belgium. In 2003 another 22 beavers from Bavaria were reintroduced to ‘Flanders’ [26]. Since 2000, beavers have recolonised Luxembourg by natural resettlement from bordering countries. A relict population survived the big European bottleneck at the lower Rhône valley (*C. f. galliae*) in France. From this population 273 beavers were released during 26 reintroduction projects in France [27]. A detailed population status can be checked in Dewas *et al.* [28].

Region V is the only area in this study, where North American beaver (*C. canadensis*) has been detected since 2006 [28]. *C. canadensis* is present in the Eifel region in Germany, Luxembourg and Belgium. The origin of these individuals is uncertain. Illegal reintroduction and/or escapes from a zoo in Rhineland-Palatinate are discussed [21,28].

**Supporting References**

1. Halley DJ, Rosell F (2002) The beaver's reconquest of Eurasia: status, population development and management of a conservation success. Mammal Rev 32: 153-178.

2. Siek J (2012) Kartierung der Biber in Hessen im Jahr 2012. Germany: Regierungspräsidium Darmstadt.

3. Schmidbauer M (2012) Biber in Unterfranken. Germany: Regierung von Unterfranken.

4. Dolch D, Heidecke D, Teubner J, Teubner J (2002) Der Biber im Land Brandenburg. Naturschutz und Landschaftspflege in Brandenburg 11: 220-234.

5. Gozdziewski J, Gizejewski Z (2012) Current status of the European beaver population in Poland. In: Bjedov L, Schwab G, Grubesic M, editors. 6th international Beaver Symposium. Ivanic-Grad, Croatia: Faculty of Forestry, University of Zagreb. pp. 81.

6. Graczyk R (1981) Zur Wiedereinbürgerung des Bibers in der Kulturlandschaft Polens. Jagd u Hege St Gallen 13: 34-35.

7. Gizejewski Z, Raczek-Zakrzewski M (2012) Farm breeding of European beaver (*Castor fiber* L.) in the Research Station of the Polish Academy of Sciences at Popielno. In: Bjedov L, Schwab G, Grubesic M, editors. 6th International Beaver Symposium. Ivanic-Grad; Croatia: Faculty of Forestry, University of Zagreb. pp. 81.

8. Djoschkin WW, Safonow WG (1972) Biber der alten und neuen Welt; Ziemsen Verlag. Wittenberg-Lutherstadt: Neue Brehm Bücherei.

9. Schaper F (1976) Wiedereinbürgerung von Bibern – Entwicklung einer Biberkolonie bei Nürnberg. Mitteilungen der Zoologischen Gesellschaft Braunau 2: 281-342.

10. Waechter A (1972) Le Castor en Alsace: Lien ornithol. d`Alsace, Straßbourg.

11. Vogel R (1941) Die alluvialen Säugetiere Württembergs. Jh Verh Vaterl Naturk Württ 96: 89-112.

12. Wegele L (1963) Vom Biber in Schwaben. Ber Naturwiss Ver Schwaben EV 67: 29-59.

13. Weinzierl H (1973) Projekt Biber. Wiedereinbürgerung von Tieren: Kosmos.

14. Rouland P (1991) La réintroduction du Castor en France. Courner de la Cellule Environnement de l`INRA 14: 35-42.

15. Auer W (1976) Ein Beitrag zur Wiedereinbürgerung des Bibers in Baden-Württemberg. Mitteilungen der Zoologischen Gesellschaft Braunau 3: 16-20.

16. Rieder N, Rohrer P (1982) Über die Möglichkeit der Wiederansiedlung des Bibers (*Castor fiber* L.) in Südwestdeutschland. Carolinea 40: 91-98.

17. Stocker G (1985) Biber (*Castor fiber*) in der Schweiz. Probleme der Wiedereinbürgerung aus biologischer und ökologischer Sicht. Eidg Anstalt für das forstliche Versuchswesen, Birmensdorf: 149.

18. Allgöwer R (2005) Der Biber - *Castor fiber*. In: Braun M, Dieterlen F, editors. Die Säugetiere Baden-Württembergs: Ulmer Verlag. pp. 181-189.

19. Rahm U, Bättig M (1996) Der Biber in der Schweiz. Bestand, Gefährdung. Schutz. Bundesamt für Umwelt, Wald und Landschaft (BUWAL). 68 p.

20. Angst C (2010) Mit dem Biber leben. Bestandeserhebung 2008. Perspektiven für den Umgang mit dem Biber in der Schweiz. Umwelt-Wissen Nr 1008 Bundesamt für Umwelt, Bern, und Schweizer Zentrum für die Kartographie der Fauna, Neuenburg: 156.

21. Schley L, Herr J, Dalbeck L, Denné R, Manet B, et al. (2009) Evidence for the presence of the North American beaver *Castor canadensis* in Western Europe. 5th International Beaver Symposium. Dubingiai, Lithuania. pp. 58.

22. Schneider E, Schulte R (1985) Befunde zu den Habitatansprüchen des Europäischen Bibers *Castor*

*fiber* L. aus einem Wiederansiedlungsveruch an einem Mittelgebirgsbach der nördlichen Eifel.

Zeitschrift für angewandte Zoologie 72: 161–179.

23. Naumann G (1991) Aussetzen von Bibern in der Eifel – Ein Beispiel für die Problematik von Wiedereinbürgerungen. In: Rheinischer Verein für Denkmalpflege und Landschaftsschutz, editor. Rheinischer Verein für Denkmalpflege und Landschaftsschutz. Neuss, Deutschland. Yearbook 1989–1991: 137–150

24. Bünning I, Bräsecke R, Geiger-Roswora D (2004) Biber (*Castor fiber*) in Nordrhein-Westfalen. LÖBF-Mitteilungen 3: 52-58.

25. Schwab G, Schmidbauer M. The Bavarian beaver re-introductions. In: Czech A, Schwab G, editors. The European Beaver in a New Millennium; 2002; Krakow, Poland. Carpathian Heritage Society. pp. 51–53.

26. Verbeylen G (2003) The unofficial return of the European beaver (*Castor fiber*) in Flanders (Belgium). Lutra 462: 123-128.

27. Rouland P, Migot P (1990) La réintroduction du castor (*Castor fiber*) en France. Revue d’Ecologique la Terre et la Vie 5: 145–158.

28. Dewas M, Herr J, Schley L, Angst C, Manet B, et al. (2012) Recovery and status of native and introduced beavers *Castor fiber* and *Castor canadensis* in France and neighbouring countries. Mammal Rev 42: 144-165.
